# Spatial and temporal modulation of enterotoxigenic *E. coli* H10407 pathogenesis and interplay with microbiota in human gut models

**DOI:** 10.1186/s12915-020-00860-x

**Published:** 2020-10-14

**Authors:** Charlène Roussel, Kim De Paepe, Wessam Galia, Jana De Bodt, Sandrine Chalancon, Françoise Leriche, Nathalie Ballet, Sylvain Denis, Monique Alric, Tom Van de Wiele, Stéphanie Blanquet-Diot

**Affiliations:** 1 Université Clermont Auvergne, UMR UCA-INRA 454 MEDIS, Microbiology Digestive Environment and Health, Clermont-Ferrand, France; 2 CMET, Center for Microbial Ecology and Technology, Department of Biotechnology, Faculty of Bioscience Engineering, Ghent University, Ghent, Belgium; 3 UMR 5557 Microbial Ecology, Research Group on Bacterial Opportunistic Pathogens and Environment, CNRS, VetAgro Sup, Lyon, France; 4 Unité de recherche Fromagère, VetAgro Sup, Lempdes, France; 5 Lesaffre International, Lesaffre Group, Marcq-en-Baroeul, France

**Keywords:** ETEC pathogenesis, Survival, Virulence, Gut microbes, Digestive in vitro systems

## Abstract

**Background:**

Enterotoxigenic *Escherichia coli* (ETEC) substantially contributes to the burden of diarrheal illnesses in developing countries. With the use of complementary in vitro models of the human digestive environment, TNO gastrointestinal model (TIM-1), and Mucosal Simulator of the Human Intestinal Microbial Ecosystem (M-SHIME), we provided the first detailed report on the spatial-temporal modulation of ETEC H10407 survival, virulence, and its interplay with gut microbiota. These systems integrate the main physicochemical parameters of the human upper digestion (TIM-1) and simulate the ileum *vs* ascending colon microbial communities and luminal *vs* mucosal microenvironments, captured from six fecal donors (M-SHIME).

**Results:**

A loss of ETEC viability was noticed upon gastric digestion, while a growth renewal was found at the end of jejunal and ileal digestion. The remarkable ETEC mucosal attachment helped to maintain luminal concentrations above 6 log_10_ mL^−1^ in the ileum and ascending colon up to 5 days post-infection. Seven ETEC virulence genes were monitored. Most of them were switched on in the stomach and switched off in the TIM-1 ileal effluents and in a late post-infectious stage in the M-SHIME ascending colon. No heat-labile enterotoxin production was measured in the stomach in contrast to the ileum and ascending colon. Using *16S* rRNA gene-based amplicon sequencing, ETEC infection modulated the microbial community structure of the ileum mucus and ascending colon lumen.

**Conclusions:**

This study provides a better understanding of the interplay between ETEC and gastrointestinal cues and may serve to complete knowledge on ETEC pathogenesis and inspire novel prophylactic strategies for diarrheal diseases.

## Background

The food and waterborne enterotoxigenic *Escherichia coli* (ETEC) is one of the major etiological agents of traveler’s diarrhea and infant diarrhea in the world [1]. Strongly associated with poor hygiene facilities, ETEC has chiefly affected low-income civilizations in south Asia, Africa, and Latin America. An estimated 44 million of ETEC-related diarrheal diseases occur annually, resulting in 113,000 deaths in 2015 [2, 3].

Once ingested at a dose of 10^8^ to 10^10^ cells in adults [4], ETEC pursues a sophisticated strategy to withstand the stringent factors encountered in successive gastrointestinal niches (e.g., acidic pH, bile acids, antimicrobial peptides, and gut microbes) [1]. In the distal part of the small intestine, ETEC effectively penetrates the mucus layer through mucin-degrading proteins, promoting ETEC attachment to the intestinal epithelial cells [5]. Such adhesion is orchestrated by a primary set of fimbrial (e.g., CFA, FimH), non-fimbrial (e.g., Tia) adhesins, and accessory colonization factors like EtpA. The adhesion will facilitate the production and delivery of heat-labile (LT) and/or heat-stable (ST) enterotoxins, the hallmark of ETEC pathogenesis [6]. LT excretion in the intestinal lumen is mediated by the protein labile enterotoxin output (LeoA) and type-2 secretion system, while ST excretion is mediated through the TolC channel [6, 7]. Upon release and binding in the small intestine, LT and/or ST enzymatic activity results in the opening of cystic fibrosis transmembrane regulator which creates an osmotic movement of water into the intestinal lumen, leading to profuse watery diarrhea.

So far, the underlying mechanisms of ETEC pathogenesis through its survival and virulence in the human gastrointestinal tract remain scarcely understood. Although ethically constrained, the use of wild and attenuated ETEC in human challenges is suitable in order to follow the onset of symptoms [8]. However, to assess ETEC behavior throughout the human gut, these trials are ethically and practically not appropriated since they require a dynamic sampling of the gastrointestinal content. As a means of recourse, ETEC findings predominantly originate from animal models, intestinal epithelial cell cultures, and simple static in vitro models of the human gastrointestinal tract. All these approaches are respectively limited by clear differences between animal and human gut physiology, the ignorance of successive gastrointestinal niches encountered by the pathogen prior to host/cell interactions, and the simplicity of in vitro models simulating only one digestive parameter at a time. In addition, only three recent studies have explored the human gut microbiota changes during the initiation and progression of ETEC diarrhea [9–11]. No data are available on how the human gut microbiota may influence ETEC survival and virulence. The few studies available all relied on fecal samples and rectal swabs collected from patients that experienced an ETEC episode, or in the course of a clinical trial where healthy adults have been challenged with an infra-physiological dose of ETEC [9–11]. However, it is generally accepted that fecal microbial communities fundamentally differ from microbial communities residing in other locations in the gastrointestinal tract.

**Fig. 1:**
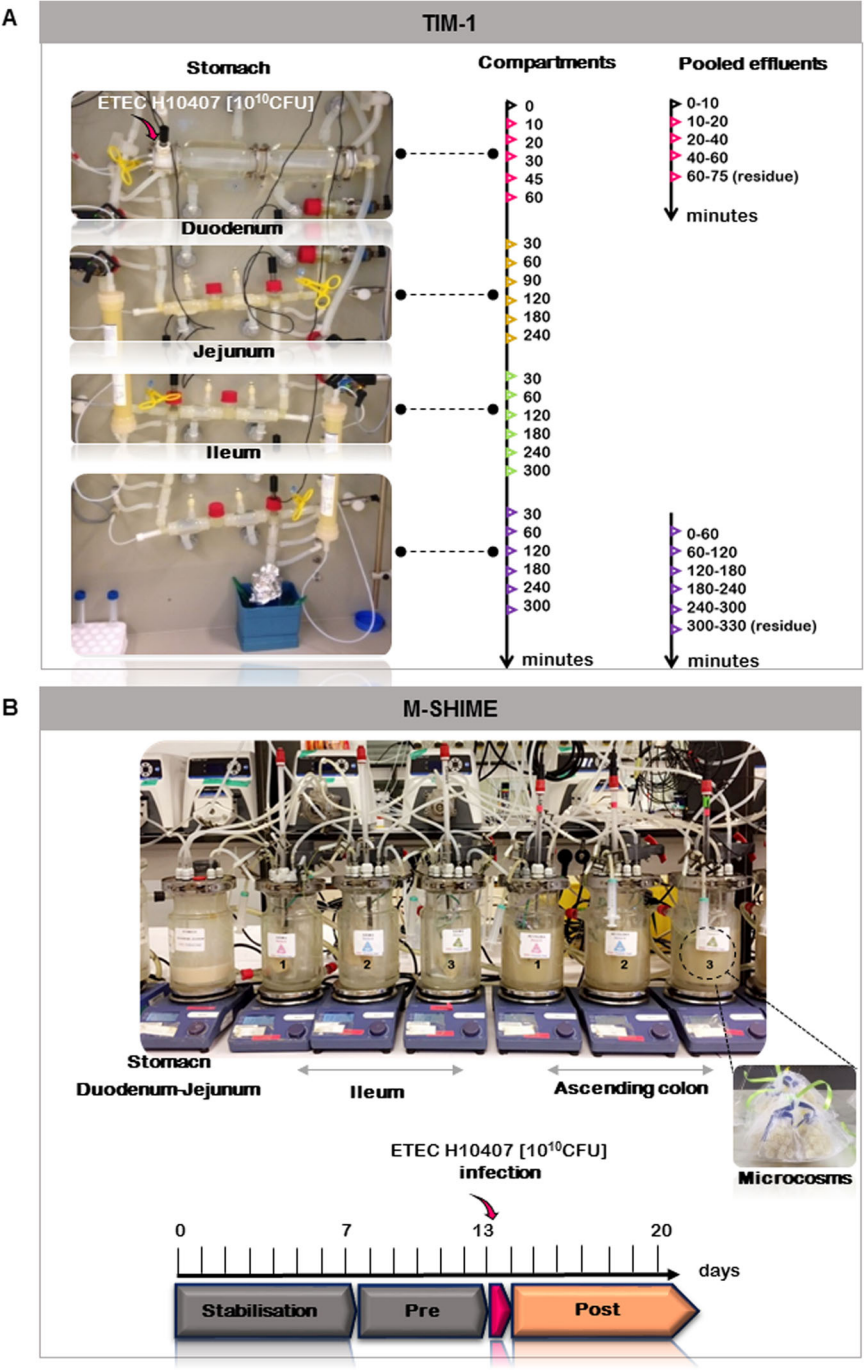
TIM-1 and M-SHIME set-up of the present study. **a** On the left side, picture of the TIM-1 system mimicking the main physicochemical parameters of the gastro-intestinal digestion (gastric pH, digestive secretion delivery, transit time, and passive absorption). Experiments were reproducing an adult ingesting a glass of water contaminated with ETEC. On the right side, sampling times (minutes) associated to the gut regions. Samples were taken directly in each compartment; or indirectly by pooling the gastric effluents when the stomach compartment was solely used, or the ileal effluents when the entire system was used. **b** Picture of the M-SHIME system mimicking the digestive and fermentative conditions of three individuals. The run has been performed twice (six distinct individuals). The stomach/combined duodenum-jejunum vessel was connected with three ileum bioreactors coupled to three ascending colon vessels. Upon start-up (day 0), the ascending colons were inoculated with the fecal samples obtained from three individuals. The fecal inoculation of the ileum started at day 3, by introducing a small amount of the fecal suspension collected in the ascending colon for each individual. To reproduce the mucosal phase, microcosms coated with type III mucin-agar were introduced in each ileum and ascending colon vessels. ETEC infection was performed at day 13 in the ileum. Samples were taken daily during the 20 days fermentation

Therefore, integration and sequential delivery of gastrointestinal signals are needed to model the dynamics and complexity of the human gut more closely as well as to investigate ETEC behavior in different gastrointestinal environments. In particular, simulation of the gastric pH drop, representative gastrointestinal transit time, and reproduction of a highly complex gut microbiota from human origin are some of the key parameters required to strengthen the conclusion of previous studies. Regionalized and dynamic in vitro models are valuable alternatives to fully assess pathogenic strains and more closely approximate the complexity of the human gastrointestinal physiology. Among the available systems, the multi-compartmental and computer-controlled TNO gastrointestinal model (TIM-1) is currently considered as the most complete simulator of the upper gastrointestinal tract by simulating the main physicochemical parameters of the human digestion [12, 13] (Fig. 1a). Similarly, the Mucosal Simulator of the Human Intestinal Microbial Ecosystem (M-SHIME) is the most complete multistage system of the lower gastrointestinal tract, cultivating a complex gut microbiota under controlled environmental conditions and mimicking the main abiotic factors of colonic fermentation process [14] (Fig. 1b). Those in vitro systems have been well-validated by in vitro/in vivo correlation studies [15–17].

In the present study, we operated the complementary in vitro gut models TIM-1 and M-SHIME in order to unravel the mechanisms associated with ETEC H10407 pathogenesis in the human gastrointestinal tract and to monitor the regionalized gut microbial succession following ETEC infection. Hence, this study greatly contributes to assessing the dynamics of ETEC survival, physiological state, and its virulence features, in successive gut niches that the pathogen encounters in humans. So far, our work represents the most complete survey of ETEC pathogenesis in the mimicked human gastrointestinal ecosystem.

## Results

### Gastric acid prevents ETEC survival while small intestinal conditions help promoting its growth renewal at the end of gastrointestinal digestion

**Fig. 2:**
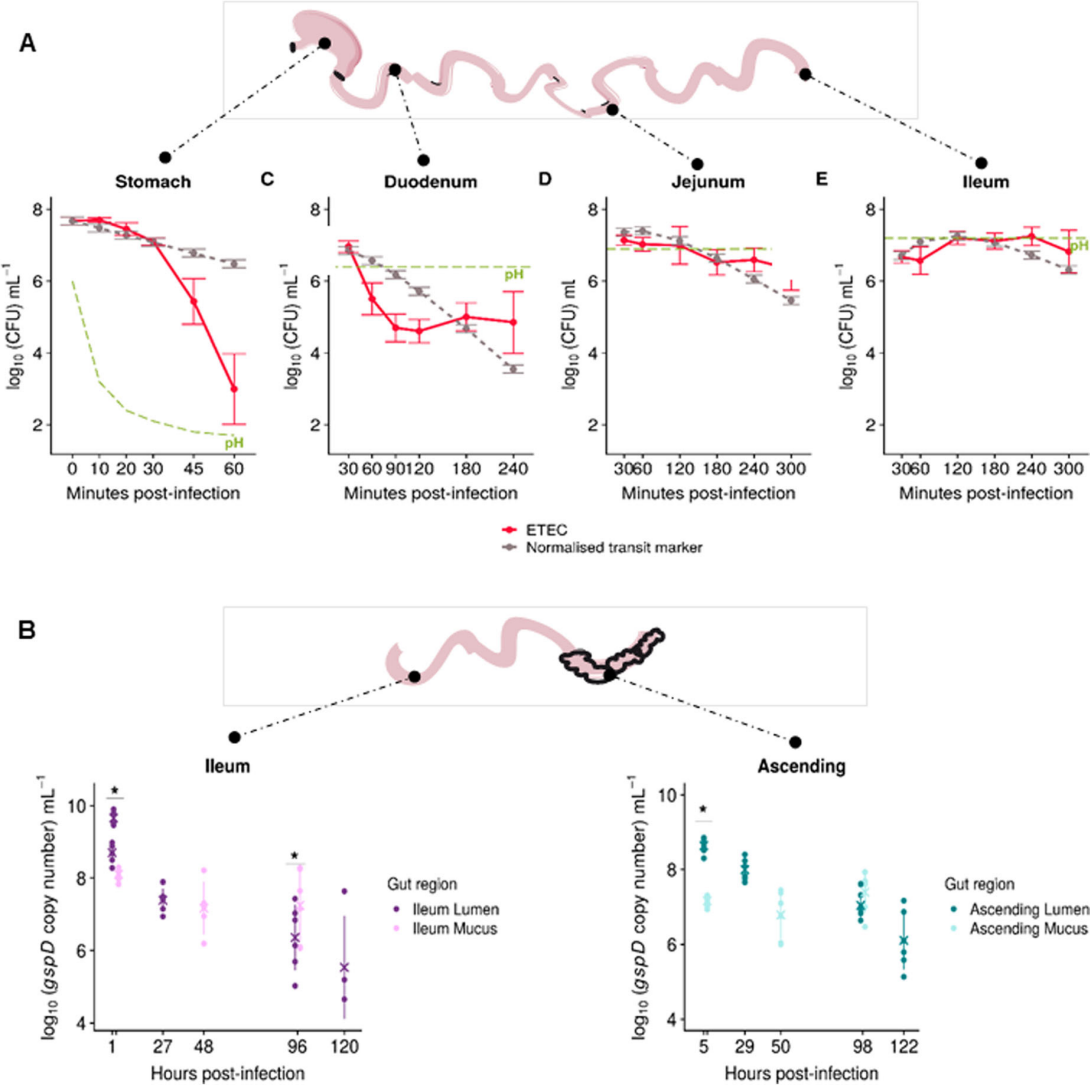
Dynamics of ETEC survival in different gastrointestinal regions of the TIM-1 and M-SHIME systems. **a** After introduction of a glass of ETEC-contaminated water in the TIM-1 stomach, the number of cultivable ETEC cells in each compartment was determined by plate counts. Results are expressed as mean concentrations in log_10_ CFU mL^−1^ ± SD of four independent replicates (red line), compared with an inert and non-absorbable transit marker indicating 100% survival (gray dashed line). Bacterial curves below that of the transit marker reflect cell mortality, while curves above the transit marker are indicative of bacterial growth. The level of pH in each compartment is illustrated in green. No statistical significant differences were found between ETEC and transit marker kinetics. **b** After gastro-jejunal digestion of a glass of ETEC-contaminated water under static batch, the pre-digested ETEC inoculum was introduced in the M-SHIME ileum at day 13 and followed up till 122 h post-infection in the luminal and mucosal phases of the ileum and ascending colon. ETEC survival was estimated by qPCR and expressed in log_10_ *gspD* copy number mL^−1^ ± SD of six different healthy donors. Statistically significant differences between ETEC survival in the luminal *vs* the mucosal phases are denoted at *p* < 0.05 (*), as determined by pairwise Wilcoxon rank sum tests with Holm correction

The TIM-1 was used to simulate the effect of physicochemical parameters and upper gastrointestinal transit on ETEC survival (Fig. 2a). At time point T0, a glass of ETEC-contaminated water (7.7 ± 0.1 log_10_ mL^−1^) was introduced into the stomach compartment. During in vitro digestion, regionalized kinetics of ETEC survival were compared to a non-absorbable inert transit marker, representing a 100% survival (Fig. 2a). In the gastric compartment, a loss of ETEC viability was observed from 45 min digestion and associated to a rapid real-time pH drop. ETEC remained at 2.9 ± 0.9 log_10_ mL^−1^ at the end of gastric digestion (T60 min). In the duodenum, ETEC concentrations gradually decreased until 120 min digestion and tended to recover from 180 min. Conversely, in the jejunum and ileum, ETEC kinetics paralleled that of the transit marker during the first 180 min of digestion. A slight re-growth was afterwards observed in both gut regions. ETEC reached 6.6 ± 0.6 log_10_ mL^−1^ and 7.3 ± 0.3 log_10_ mL^−1^ at the end of the jejunal (T300 min) and ileal (T240 min) digestion, respectively (Fig. 2a). At the end of the upper gastrointestinal digestion, a global ETEC survival percentage of 65 ± 31% was found in pooled gastrointestinal effluents (Additional file 1: Fig. S1).

### Mucosal niches harbor ETEC’s colonization in ileum and ascending colon

The impact of the ileal and ascending colon environments on ETEC colonization was assessed in the M-SHIME (Fig. 2b). To account for inter-individual variability in gut microbiota composition and metabolic activity, microbial communities deriving from six healthy human individuals were tested. On top of the luminal microenvironments, the intestinal mucus layer was reproduced in both ileum and ascending colon compartments. Given the complex microbial background, the *gspD* gene was used to characterize dynamics of ETEC survival in the M-SHIME. ETEC suspensions obtained after static gastro-jejunal digestion were inoculated in the SHIME ileum (8.8 ± 0.3 log_10_ mL^−1^ *gspD* copy number) (Fig. 2b). ETEC concentrations were significantly higher in luminal *vs* mucosal phases in ileum and ascending colon 1 h (*p =* 0.03), respectively, 5 h post-infection (*p =* 0.03), as determined by pairwise Wilcoxon rank sum tests with Holm correction. In contrast, at a later stage in the ileum (96 h post-infection), ETEC *gspD* copy numbers were significantly higher in mucosal *vs* luminal phase with 7.3 ± 1.0 log_10_ mL^−1^ compared to 6.8 ± 0.9 log_10_ mL^−1^ (*p =* 0.03). In the ascending colon (98 h post-infection), luminal and mucosal niches did not display a significant difference of ETEC *gspD* copy numbers and remained above 7 log_10_ mL^−1^ (*p =* 0.18). Overall, the remarkable ETEC mucosal attachment helped to maintain luminal concentrations above 6 log_10_ mL^−1^ until the end of the follow-up period, which was 120 h, respectively, 122 h post-infection in the ileum and ascending colon (Fig. 2b).

### ETEC modulates its membrane physiology according to the gastrointestinal niches

**Fig. 3:**
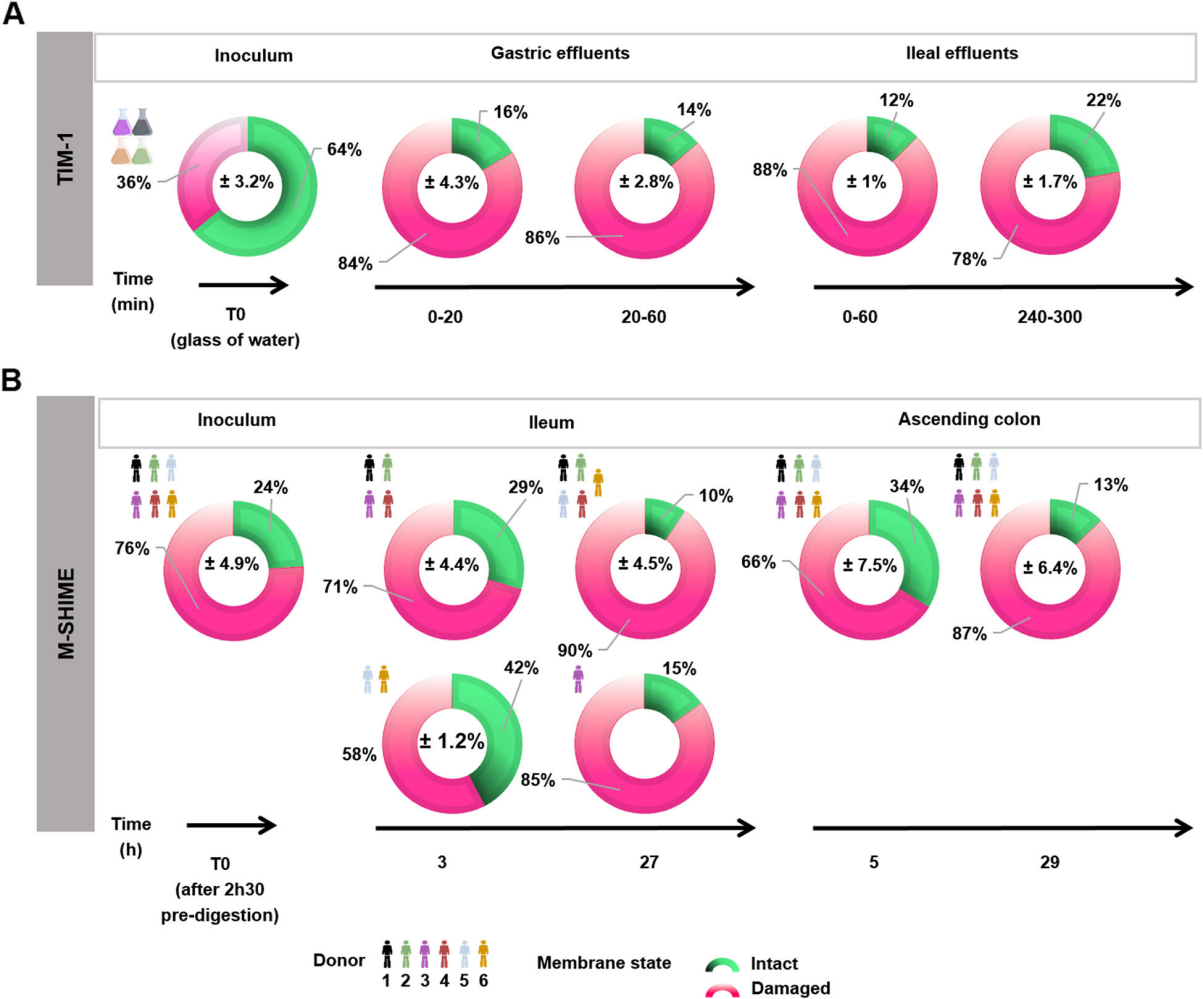
Dynamics of ETEC membrane integrity determined by PMA-qPCR in the TIM-1 and M-SHIME. ETEC membrane integrity was estimated by PMA-qPCR (*Enterobacteriaceae 16S* rRNA and ETEC *gspD* genes for TIM-1 and M-SHIME samples, respectively) and expressed as the average percentage of intact (green) and damaged (shade of pink) ETEC cells. Damaged ETEC cells were obtained after deducting the number of intact cells from the total ETEC cells measured by qPCR. ETEC membrane integrity was determined over time: **a** in the inoculum, gastric and ileal effluents of the TIM-1 (*n* = 4) and **b** in the inoculum, ileum, and ascending colon of the M-SHIME (*n* = 6). When inter-individual variability was important, a second doughnut was represented

To further investigate the effect of gastrointestinal digestion on ETEC pathogenesis, the physiology of ETEC membranes was monitored in the TIM-1 by propidium monoazide (PMA)-qPCR (Fig. 3a) and Live/Dead flow cytometry (Additional file 1: Fig. S2). Two subpopulations were discriminated by PMA-qPCR (Fig. 3a) while four ETEC membrane states were distinguished with flow cytometry by gating on the cytogram (Additional file 1: Fig. S2A). At the initial time T0, 64% of the population was detected as viable (Fig. 3a). During gastric digestion, the ETEC fraction with intact membranes decreased over time, indicating that most of the cells reaching the small intestine were damaged (Fig. 3a, Additional file 1: Fig. S2B). This result was in accordance with a notably low ETEC intracellular pH (pHi) in the gastric compartment (Additional file 1: Table S1) and a membrane depolarization at 60 min in the gastric effluents (Additional file 1: Table S2). In the ileal effluents, reflecting all environments crossed by ETEC along the gastric then intestinal digestion, flow cytometry analysis displayed a restoration of ETEC membrane physiology and the return to a slight alkaline pHi as well as a membrane polarization (Additional file 1: Fig. S2B, Tables S1–2). ETEC membrane restoration was less evident with PMA-qPCR analysis since the 78% displayed at 300 min represents a mix of both dead and damaged cells (Fig. 3a). Consequently, at the end of the gastrointestinal digestion, nearly 1/4 of the total ETEC cells entering the colon had an intact membrane (Fig. 3a, Additional file 1: Fig. S2B).

In the M-SHIME, the use of flow cytometry was impossible due to the complex microbial background. PMA-qPCR was the sole technique employed to track ETEC viability in the luminal phase (Fig. 3b). Three hours post-infection in the ileum and 5 h post-infection in ascending colon, the number of viable ETEC tended to increase in comparison with pre-digested ETEC inoculum containing approximately 24% living cells (*p =* 0.03), as determined by pairwise Wilcoxon rank sum tests with Holm correction. The ratio of intact ETEC cells progressively decreased along fermentation with an important donor variability in the ileum. In the ascending colon, all donors displayed a low number (13%) of viable ETEC 29 h post-infection (Fig. 3b).

### ETEC virulence gene expression profiles are affected by gastrointestinal passage in the TIM-1 and niche-specific gut environments in the M-SHIME

**Fig. 4:**
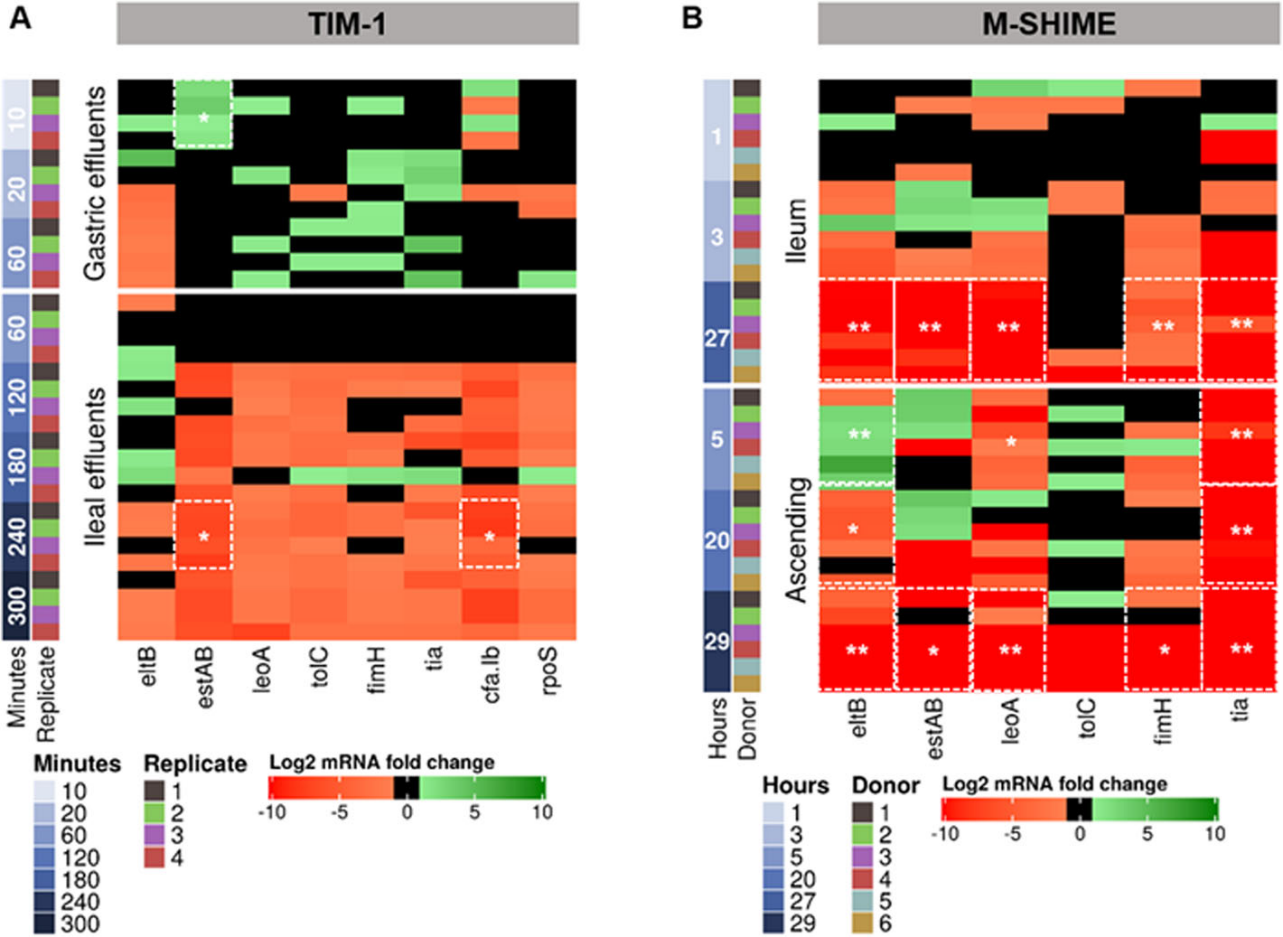
Dynamics of ETEC virulence gene expression in different gastrointestinal regions of the **a** TIM-1 and **b** M-SHIME. Results were determined by RT-qPCR, expressed and colored according to the log_2_ fold-change. For reasonable statements, statistically significant differential expression had to meet two criteria: a mean log_2_ fold change expression of ≥ 1 (induction denoted in shade of green) or ≤ − 1 (repression denoted in shade of red) and a *p* ≤ 0.05*.* The statistically significant differences in average of log_2_ gene expression at each time points for the four (TIM-1) or six (M-SHIME) replicates were compared to the initial inoculum (T0) and marked by the white frames in the heatmap, as determined by the Friedman post hoc Nemenyi test

The expression of the main ETEC H10407 virulence genes encoding for attachment and colonization (*cfa/Ib*, *tia*, *fimH*), enterotoxin production (*eltB*, *estP*), and release of enterotoxins (*leoA*, *tolC*) within the host was followed up over time in the TIM-1 and luminal phase of the SHIME. The log_2_ fold change of each gene at any time point was thereto compared with ETEC T0 (in a glass of water prior to gastric digestion in TIM-1 *vs* ETEC T0 after pre-gastro-jejunal digestion in batch prior to introduction in M-SHIME ileum), and results are shown for each replicate individually (Fig. 4, Additional file 1: Tables S3–4).

In the TIM-1, the virulence gene expression profiles were more induced in gastric effluents and repressed in ileal effluents, particularly from 120 min digestion (Fig. 4a). Two distinct expression patterns were discerned for the enterotoxins *eltB* and *estP* in both gastric and ileal effluents. The mean expression of *estP* significantly increased by 2.2 ± 1.0 log_2_ fold (*p =* 0.03) upon exposure to gastric conditions in the first 10 min (pH 3.2), while *eltB* expression was unchanged, as determined by the Friedman post hoc Nemenyi test. Then, from 20 to 60 min digestion, *eltB* tended to be repressed and positively correlated with low pH (Additional file 1: Fig. S3A) while *estP* expression was unchanged compared with T0 (Fig. 4a). In the ileal effluents, *eltB* tended to be induced in half of the replicates in the first 180 min of the digestion. *estP* was repressed from T120 to T300 min in the ileal effluents, with a significant − 7 ± 0.6 log_2_ fold repression at T240 min (*p =* 0.05). The two genes *estP* and *cfa/Ib* displayed similar expression profiles in ileal effluents (Fig. 4a, Additional file 1: Fig. S3B). Then, *leoA* and *tolC* were expressed at a low level in gastric effluents and tended to be under-expressed in ileal effluents. The *fimH* gene encoding for type 1 pili was slightly over-expressed in gastric effluents at T20, as for *tia* (Fig. 4a). Surprisingly, *rpoS* which is known to be activated in response to environmental stresses was not over-expressed at low pH in the stomach.

The same set of ETEC virulence genes (except *rpoS*) was analyzed in the ileum and ascending colon lumen of six distinct donors (Fig. 4b). In comparison with the T0, the virulence genes were significantly (*p =* 0.01) under-expressed at late time points of fermentation in both gut regions (T27 and 29 h post-infection). The enterotoxin encoding genes *eltB* and *estP* displayed a somewhat similar expression profile but with donor-dependent differences. *eltB* was significantly over-expressed 5 h post-infection in the colon in five donors with a 2- to 3-fold log_2_ induction in donors 2, 3, 4, and 6 and a 7-log_2_ fold induction in donor 5. In the ascending colon, *leoA* was predominantly repressed over time. *tolC* expression did not substantially change compared to T0, especially in the ileum. In contrast, *tia* expression was consistently repressed in the ascending colon (Fig. 4b). *cfa/Ib* mRNA was not amplified either in the ileum and colon. Finally, correlations between genes were mostly found in the ileum compared to the ascending colon (Additional file 1: Fig. S3C, D).

### LT production is undetected in the stomach and maximized in ascending colon despite high donor variability

**Fig. 5:**
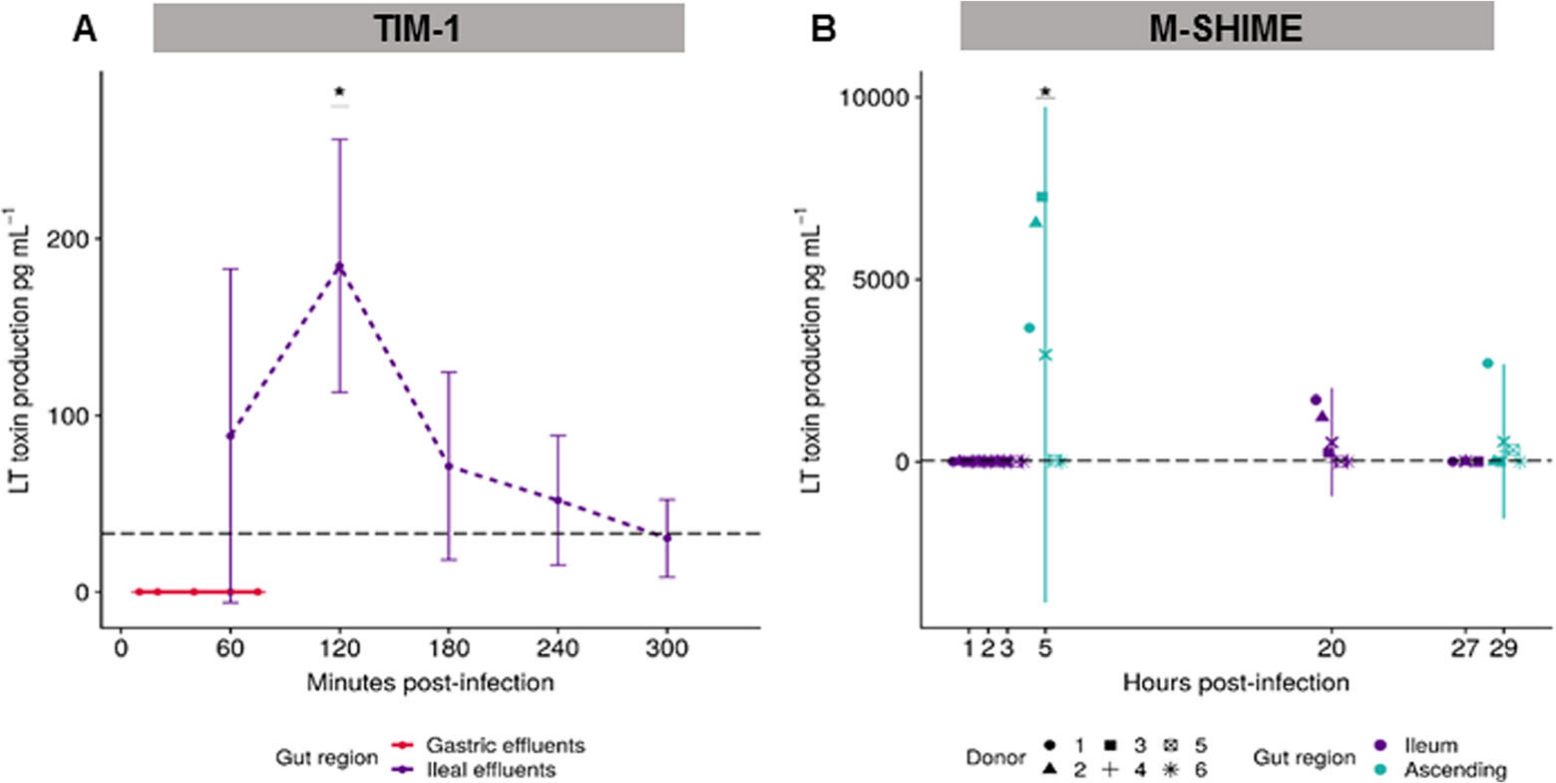
Dynamics of LT toxin production in different gastrointestinal regions of the TIM-1 and M-SHIME. Results were determined by GM1 ELISA. LT toxin production was expressed as mean pg mL^−1^ ± SD of **a** four independent replicates in the gastric and ileal effluents of the TIM-1 and **b** six different donors (denoted by the different shapes) in the luminal ileum and ascending colon of the M-SHIME. The horizontal black dashed line represents the detection limit. Statistically significant differences in LT toxin production over time compared to the initial inoculum (T0) are denoted at *p* < 0.05 (*), as determined by the Friedman post hoc Nemenyi test

At the protein level, LT enterotoxin production was quantified in the TIM-1 and M-SHIME (Fig. 5). LT was absent from the gastric effluents, while the toxin was detected in the ileal effluents (Fig. 5a). LT production was maximal and significantly increased compared with T0 after T120 min digestion, with a mean of production of 185 ± 72 pg mL^−1^ (*p =* 0.014), as determined by the Friedman post hoc Nemenyi test. No toxin was measured in the SHIME ileum during the first 3 h post-infection in all donors (Fig. 5b); yet, LT was produced at high levels varying between 3500 to 7200 pg mL^−1^ in the ascending colon, 5 h post-infection in three (donors 1, 2, and 3) out of the six donors (*p =* 0.042). Although lower, LT production was still achieved 20 h post-infection in the ileum, by three (donors 1, 2, and 3) of the six donors (Fig. 5b). Remarkably, the donors displaying the highest *estP* gene expression level (donors 1 to 3) in the ascending colon (Fig. 4b) also produced the highest amount of the LT toxin (Fig. 5b). LT toxin production was found to be negatively correlated with ETEC survival at the end of digestion in the ileum of the TIM-1 and M-SHIME (Additional file 1: Fig. S3B, C).

### Microbes define the regional identity of the gut compartments

**Fig. 6:**
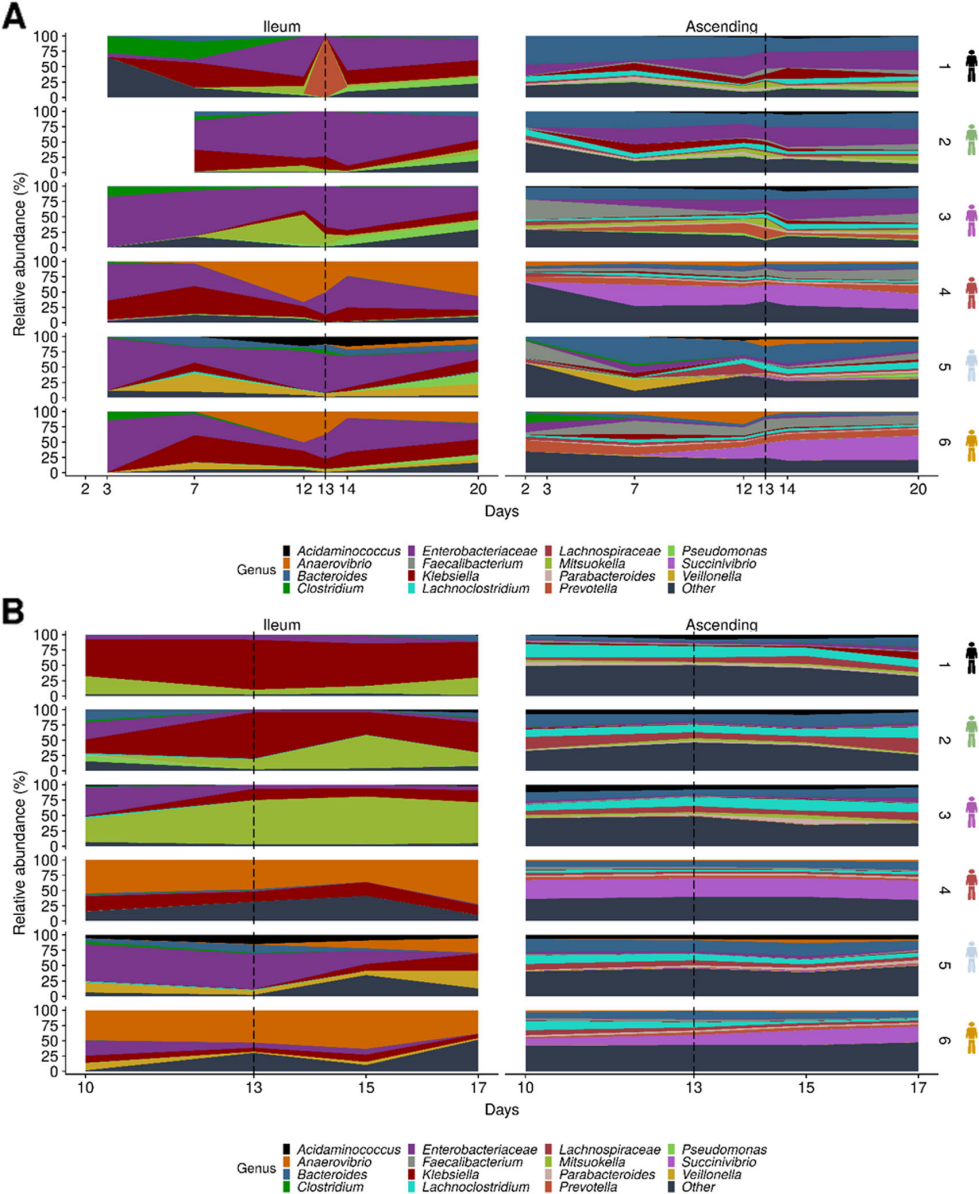
Genus level microbial community composition of the ileum and ascending colon environments in the M-SHIME. The area graphs show the relative abundance of the 15 most abundant genera in the **a** luminal and **b** mucosal ileum and ascending colon from six different donors over the course of 20 days fermentation, as determined by amplicon sequencing. ETEC infection is demarcated by the dashed line at day 13

The microbial populations in the different simulated environments of the M-SHIME were separately derived from the fecal microbiota of six donors. The experimental set-up allowed to discriminate between cross-sectional and longitudinal regions: ileum lumen, ileum mucus, ascending colon lumen, and ascending colon mucus (Fig. 6, Additional file 1: Fig. S4–6). The gut regionalization resulted in profound differences in microbiota composition, relative abundance, and Simpson α-diversity (Additional file 1: Fig. S7). At family and/or genus level, the ileum lumen was characterized by a high relative abundance of *Enterobacteriaceae* and *Klebsiella*, while donors 4 and 6 displayed a higher abundance of *Anaerovibrio* (Fig. 6a). In contrast, the ascending colon lumen was dominated by *Bacteroides*, *Faecalibacterium*, *Lachnoclostridium*, and *Prevotella* in most of the donors, except for donors 4 and 6 who displayed a high abundance of *Succinivibrio* genus (Fig. 6a). Species belonging to *Firmicutes* prevail in the mucosal niche (Fig. 6b, Additional file 1: Fig. S5). The ileum mucus was characterized by a high relative abundance of *Anaerovibrio*, *Klebsiella*, and *Mitsuokella* while increased microbe diversity was found in the ascending colon (Fig. 6b).

### Unique ETEC challenge modulates gut microbiota depending on the niche specificity

Distance-based redundancy analysis (db-RDA), a robust method for testing group dissimilarities, confirmed that “gut region” (e.g., ileum lumen, ileum mucus, ascending colon lumen, ascending colon mucus) was the predominant explanatory variable for dissimilarities in microbiota composition (19.5%, *p =* 0.001) (Additional file 1: Fig. S8A). The factor “donor” was the second most important contributor (6.5%, *p =* 0.001) (Additional file 1: Fig. S8B). Although a marked decrease of the Simpson α-diversity index was displayed in ileum lumen and mucus following ETEC infection (Additional file 1: Fig. S7), the factor “period” assessing the effect of pre- and post-infection did not profoundly shift the overall microbiota composition (0.9%, *p =* 0.53) (Additional file 1: Fig. S8C). To get rid of the total gut region dissimilarity and better evaluate the impact of ETEC challenge, the evolution of the microbial community composition preceding (days 7–12) and following ETEC infection (days 13–20) was assessed by discriminating the four gut regions (Additional file 1: Fig. S9). ETEC significantly contributed to the variation of the microbial community structure in the ileum mucus (2.6%, *p =* 0.01) and ascending colon lumen (1.6%, *p =* 0.04).

**Fig. 7:**
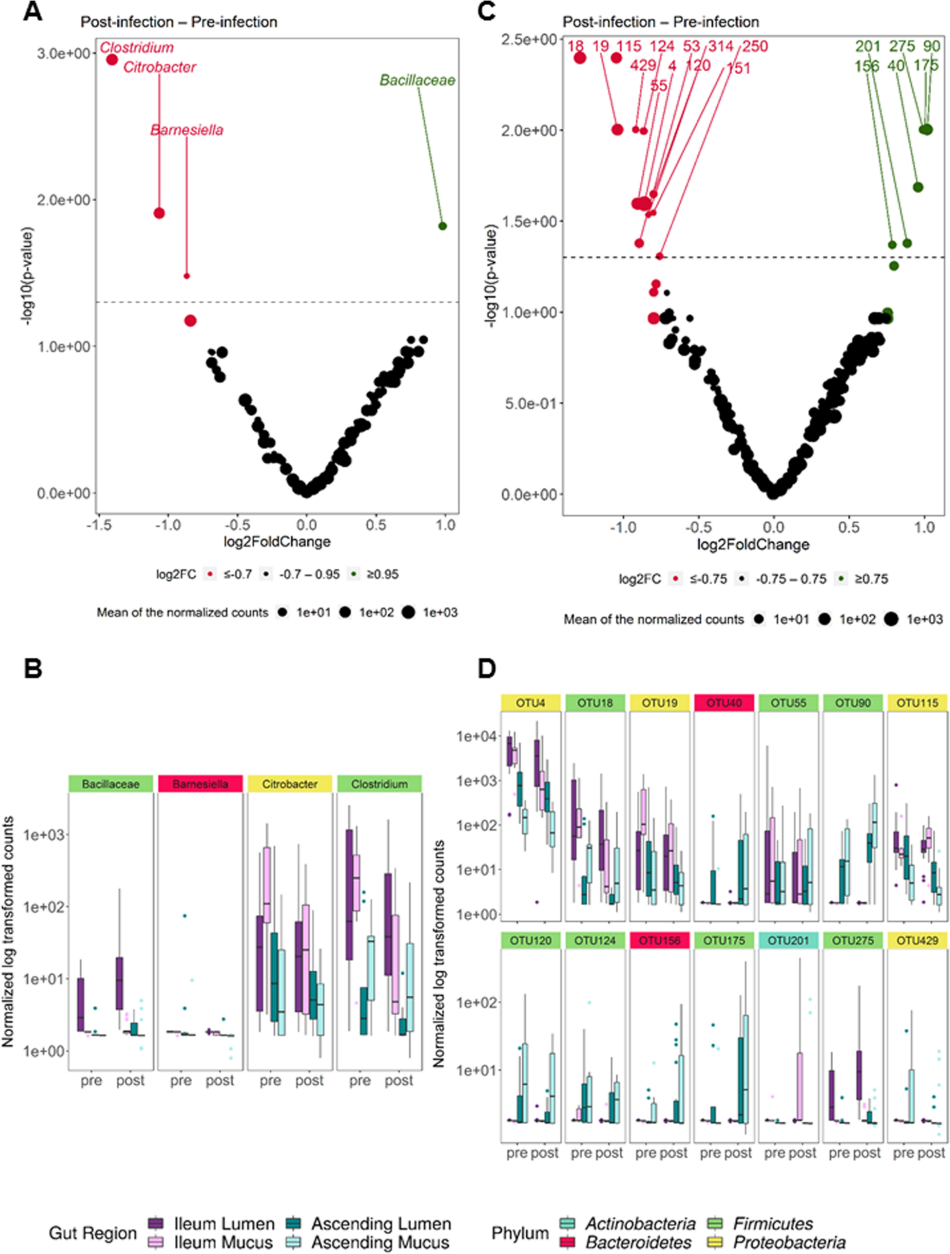
Log_2_ fold change of normalized microbial abundances between the pre- and post-infection phases in the M-SHIME. Positive log_2_ fold change indicates a stimulation of the **a** genera and **c** species in the post-infection period (in green) compared to a negative log_2_ fold change which indicates a reduction of the genera/species in the post-infection period (in red). The log-transformed adjusted *p* value is displayed on the *y*-axis and the *α* = 0.05 significance level is indicated by a dashed line. The volcano plots do not discriminate the gut regions. Significant differences in **b** genus and **d** OTU level abundance between pre- and post-infection period. The abundance is displayed for the different gut regions. Colored labels indicate the phylum classification of the respective genera and species

We further investigated the significant changes in microbiota abundance between pre- and post-infection periods in the different gut regions through a differential analysis of count data (DESeq2) (Fig. 7). Mostly, species belonging to *Firmicutes* were shifted following the ETEC challenge. In both the ileum and ascending colon, *Clostridium butyricum* (OTU18) was decreased while *Bacillus xiaoxiensis* (OTU275) as well as *Clostridium scindens* (OTU90) were stimulated in the ascending colon only (Fig. 7b, c). A few OTUs belonging to the *Proteobacteria* were decreased in the ileum and ascending colon such as OTU4 (*Enterobacter* genus), OTU19 (*Citrobacter* genus), and *Klebsiella variicola* (OTU115) except in the ileum mucus where an upsurge was observed. *Mycobacterium* (OTU201) was also remarkably increased in the ileum mucus upon ETEC supplementation. Finally, two OTUs belonging to *Bacteroidetes*, OTU40 (*Prevotella* genus), and OTU156 (*Muribaculaceae* family) were stimulated in the ascending colon following the ETEC challenge (Fig. 7b–d).

### Propionate concentration is increased following the ETEC challenge in the ileum and ascending colon

**Fig. 8:**
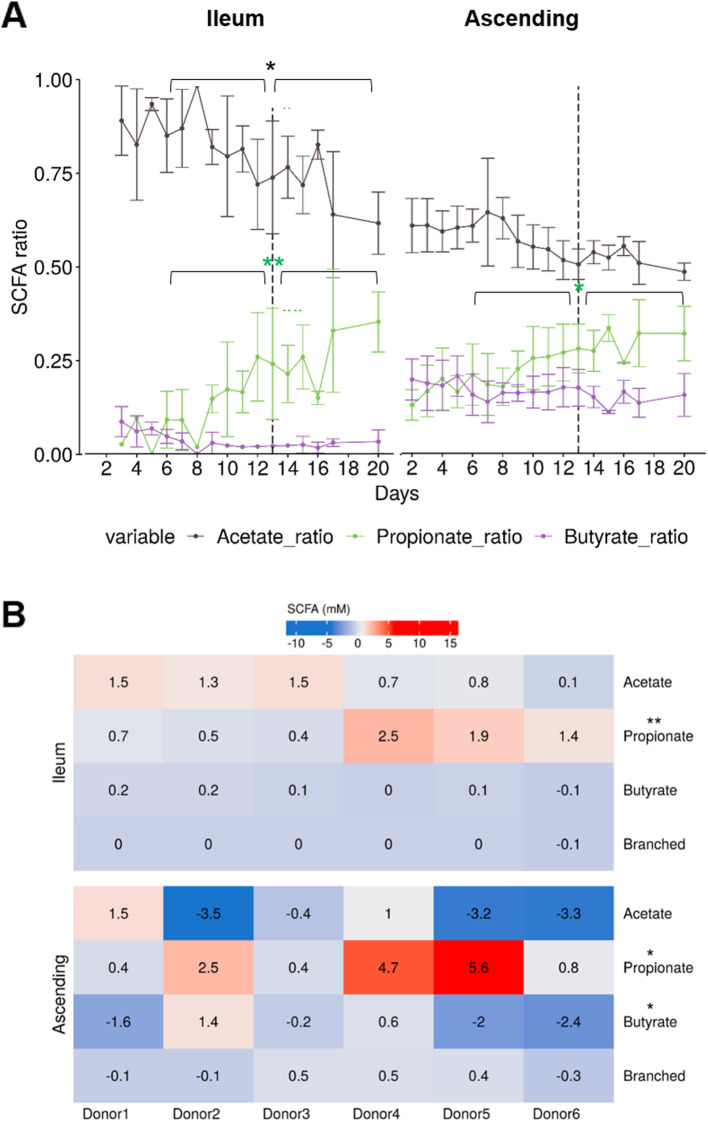
SCFA ratios and concentrations in the ileum and ascending colon compartments over time in the M-SHIME. SCFA concentrations displaying stability from day 7 to 12 were retained as the pre-infection period, and from day 13 to 20 as the post-infection period. **a** SCFA ratio was calculated as the mean acetate, propionate, and butyrate ratios of 6 donors ± SD. Statistically significant differences between pre- and post-infection periods are denoted at *p* < 0.05 (*) and *p* < 0.01 (**) as determined by pairwise Wilcoxon rank sum tests with Holm correction. **b** Difference in average concentrations of SCFA (mM) between pre- and post-infection periods in the ileum and ascending colon for the six donors. Following ETEC infection, a decrease of SCFA concentration is displayed in shade of blue while an increase is in shade of red. Statistically significant differences between pre- and post-infection periods are denoted at *p* < 0.05 (*) and *p* < 0.01 (**) as determined by pairwise Wilcoxon rank sum tests with Holm correction

The observed changes in microbial community composition between the ileum and ascending colon are also indicative of a significant change in its metabolic activity. Low SCFA concentrations were observed in the ileum compared to the ascending colon (Additional file 1: Fig. S10). ETEC infection had an impact on fermentation activity in both gut regions. A significant increase of the average propionate ratio was observed in both gut regions (ileum *p =* 0.007; ascending colon *p =* 0.01), while a decrease of the average acetate ratio (*p =* 0.01) only occurred in the ileum, as determined by pairwise Wilcoxon rank sum tests with Holm correction (Fig. 8a, Additional file 1: Fig. S10). The infection displayed a pronounced change in SCFA concentrations in the ascending colon, especially a significant increase of propionate for donors 2, 4, and 5 (*p =* 0.01) up to 5.6 mM (Fig. 8b). In contrast, acetate tended to decrease and butyrate concentration significantly decreased (*p =* 0.04) especially for donors 1, 5, and 6 (Fig. 8b). No change in branched short fatty acid concentration was seen. Next, we examined the existing correlations between SCFA concentrations and ETEC survival and virulence (Additional file 1: Fig. S11). No significant correlations were found in the ileum. In the ascending colon, acetate and propionate displayed significant and negative correlations with LT toxin production and *estP* gene expression (encoding for the ST toxin), while butyrate was positively correlated with LT production (Additional file 1: Fig. S11).

## Discussion

**Fig. 9:**
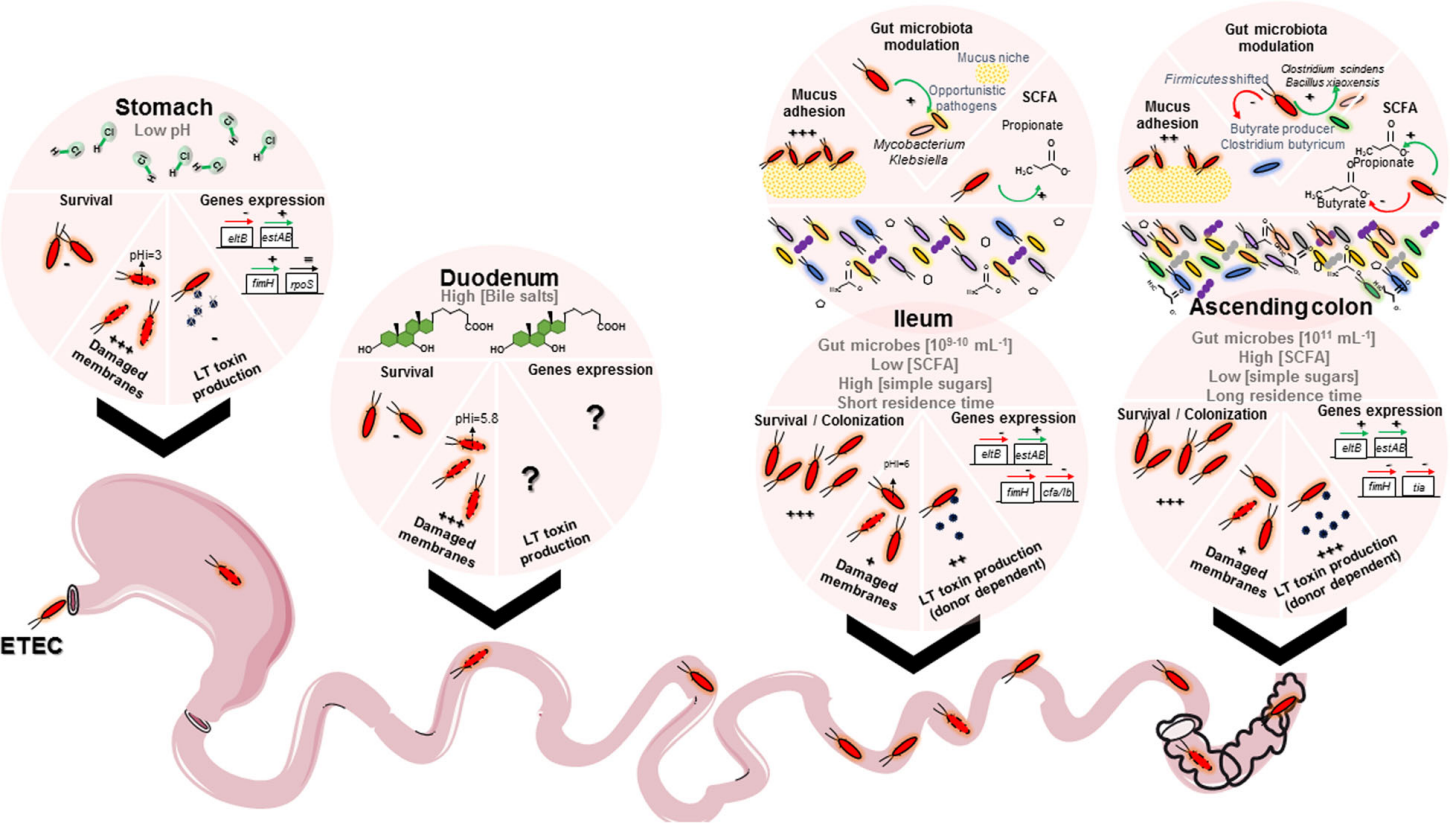
Biogeography of ETEC H10407 pathogenesis in the human in vitro systems TIM-1 and M-SHIME. The main results of the study are summed up from the stomach to the ascending colon. For each digestive compartment, the main physicochemical and/or biotic factors reproduced are indicated on the top of the circle

Interactions between ETEC bacteria, physicochemical factors, and microbiota in the human gastrointestinal tract are scarcely understood due to multiple, successive, and complex ecosystems crossed by the pathogen. With the use of validated and complementary in vitro models of the human digestive environment, TIM-1, and M-SHIME, the key objectives of the study were to unravel the dynamics of ETEC H10407 survival and virulence throughout the simulated human gastrointestinal tract from the stomach to the ascending colon. Interplay with gut microbial community and activity was also assessed in the different gut niches, i.e., ileum/ascending colon, and microenvironments, i.e., lumen/mucus. This study represents the most complete survey of ETEC pathogenesis in the mimicked human gastrointestinal ecosystem (Fig. 9).

Low gastric pH is the first challenge a pathogen faces upon ingestion. The antimicrobial effect from gastric digestion is often overestimated when static in vitro models with constant low pH are used. For instance, Masters et al. found ETEC cells to be undetectable upon 2 h static exposure at pH 2 [18]: this is not representative of healthy adult in vivo conditions. The TIM-1 model from the present study accurately captures the gradual pH drop and gastric emptying into the duodenum, resulting in ETEC being exposed to a low pH for a short time at the end of gastric digestion. Lower ETEC plate counts were hence only observed after 45 min of gastric digestion when pH dropped below 2. This drop in ETEC culturability only reveals one aspect of ETEC viability: we therefore investigated ETEC physiological states by PMA-qPCR and flow cytometry. Strikingly, we observed a sudden change of ETEC membrane integrity upon short exposure to low pH. This resulted in a high number of ETEC cells with partially compromised membranes that we assume to be related to a viable but non-culturable state [19]. This intermediate viability state temporarily prevents ETEC from growing; yet, we assume that this is a reversible effect and that most of the altered cells are resuscitated by physicochemical stimulus during passage in lower intestinal sections, as described here-after. To date, there is no effective method to distinguish culturable cells from resuscitation cells [20].

ETEC is then subjected to the duodenal conditions. The initially high levels of bile salts, an antimicrobial compound, in TIM-1’s simulated duodenum resulted in a progressive decrease of ETEC survival until halfway duodenal digestion [21]. Simulated reabsorption of bile salts and further pH increase as intestinal digestion progresses contributed to ETEC‘s increased survival in the jejunal and ileal compartments. Altogether, the number of viable ETEC was partially restored at the end of the 5-h gastrointestinal digestion with a final survival of 65% (percentage of culturable cells compared to the initial ETEC inoculum) in TIM-1 pooled ileal effluents and gastrointestinal residues.

The ileum is known to be the prime site of ETEC pathogenesis [4]. We therefore added another unique feature to this study by using an ileal M-SHIME to study ETEC’s colonization ability in the ileal environment in the presence of ileal microbiota and under anaerobiosis. Interestingly, both luminal and mucosal microenvironments maintained above 6 log_10_ mL^−1^ of ETEC up to 5 days post-infection. Intriguingly, ETEC survival in the ileal mucosa was higher than the ileal lumen. The mucus layer forms a natural habitat of intestinal symbionts and can serve as a nutrient-rich environment facilitating growth of both endosymbionts as pathogens. These observations complement our previous study where ETEC was also found to attach to mucin proteins yet in absence of microbiota [22]. While the nutritional role of the mucus layer for ETEC is unknown, it is noteworthy that ETEC’s mucosal adhesion efficacy was higher (51%) than that of the adherent invasive *E. coli* (AIEC) pathotype (20%) [23].

ETEC then transits to the colonic part. The colon is devoid of oxygen and displays a long residence time, which enhances the growth of anaerobic bacteria (10–11 log_10_ mL^−1^). It results in a high metabolic activity through the fermentation of partially digested/undigested food particles [24, 25]. Similar to our findings in the ileal environment, ETEC was able to persist and even gain a growth advantage over the luminal and mucosal colonic microbiota. Altogether, our TIM-1 and M-SHIME research demonstrated ETEC’s robustness across the entire digestive tract, even in the presence of representative physicochemical and enzymatic conditions and gut microbial populations.

The potency of ETEC to infect its host depends on virulence gene expression which is in turn determined by many gastrointestinal cues. So far, ETEC gene regulation has never been investigated in detail under realistic human upper and lower gastrointestinal conditions. We profiled not less than seven virulence genes encoding for enterotoxins (*eltB* and *estP*), enterotoxin release (l*eoA* and *tolC*), adhesins (*cfa/Ib*, *tia*, *fimH*), stress response (*rpoS*) genes, and the actual enterotoxin LT production. We found most of the virulence genes to be switched on in the stomach and switched off in the TIM-1 ileal effluents, at late post-infectious stage in the M-SHIME ileum and in the ascending colon.

We followed the *rpoS* gene expression specifically in the TIM-1. This general stress-response regulator in *E. coli* is known to contribute to the bacterial survival, for example, under acidic pH conditions. Over time, we found that the *rpoS* gene was basally expressed upon gastric digestion while under-expressed in the ileal effluents. At this stage, it remains difficult to correlate the gene expression pattern with the observed ETEC survival. In fact, most of the acid resistance system requires extracellular metabolites (e.g., glutamate, lysine) to be inducible, as well as for *rpoS* which requires polyamines [26]. In the TIM-1 stomach, the operation of such systems for ETEC was not possible due to the absence of these amino acids as only a glass of water was introduced into the system. It would be therefore of high interest to use a complex food matrix for ETEC vehicle that provides such metabolites to re-examine the regulation of *rpoS* during gastric digestion.

Upon gastric digestion, the gene *eltB* encoding LT toxin production was repressed at a pH below 3.6 and no production of LT toxin was observed. This pH-dependency for *eltB* and LT protein observed in our complex gastric in vitro system confirms previous reports from in vitro batch and in vivo (mouse) studies [27–30]. Interestingly, in the TIM-1 ileal effluents as well as in the M-SHIME ileum, no correlation was found between LT toxin production and *eltB* gene expression. On top of that, the *eltB* post-transcriptional regulations are key determinants in LT toxin production [28], and we also assumed that due to the digestive transit, toxins produced in an upper compartment can simply be found in a lower compartment without any production at this site (e.g., ileum *vs* ascending colon). The LT toxin can be associated with the lipopolysaccharide of the outer membrane of ETEC cells [30]: we therefore thought that the LT toxin can be released upon ETEC cell lysis, explaining the high production of the LT 20 h post-infection when ETEC survival decreases. However, the ETEC cell lysis observed in the stomach does not confirm this assumption, probably due to low pH. Our results also demonstrated that the LT toxin can be produced both under aerobic or anaerobic conditions, as previously shown in static cultures [31]. In comparison with *eltB*, we found that *estP* encoding for ST toxin does not follow the same expression profiles in both TIM-1 and M-SHIME. That result could be related to distinguishable gene regulation patterns. For instance, it is known that the CRP regulator represses *eltAB* transcription while it positively regulates *estP* expression [32].

We then followed up *leoA* and *tolC* encoding for the proteins contributing to secretion and delivery of enterotoxins. No significant induction of both genes was observed in the TIM-1 but *leoA* was repressed all along the M-SHIME anaerobic compartments and *tolC* had a tendency to be over-expressed in a donor-dependent manner. These results suggest that changes in environmental oxygen (TIM-1 *vs* M-SHIME) greatly influence the virulence factor expression in ETEC, as shown in a recent study comparing the levels of oxygen in batch cultures and stool samples [33]. The genes encoding for the adhesins CFA/Ib, tia, and FimH [6] have also been so far scarcely investigated. Here, we show for the first time that *fimH* and *tia* tended to be over-expressed after 20 min in the stomach, when pH is under 4. Although the underlying mechanisms are unknown, the upregulation of many fimbrial and adhesin-related genes has been reported after acidic exposure in another *E. coli* pathotype, enterohemorrhagic *E. coli* (EHEC) [12, 33]. In contrast, *fimH* and *tia* were repressed in TIM-1 and M-SHIME ileum (aerobic without microbiota *vs* anaerobic with microbiota), as well as in the ascending colon. This indicates the lack of direct roles of oxygen and microbial bulk in the modulation of *fimH* and *tia* gene expression. It therefore suggests that at the vicinity of luminal content, the presence of polarized cultured intestinal epithelium, producing apical brush borders with defined microvilli similar to intestinal human enterocyte could be required for adhesin genes induction [34]. Then, it is noteworthy that FimH acts in concert with CFA/I for optimal adhesion, as observed under in vitro Caco-2 cells assay [35]. In our study, we did not find a correlation between both gene expression patterns. *cfa/Ib* gene displayed a basal expression in the gastric environment and a repression in the TIM-1 ileum. A significant positive correlation was found between *cfa/Ib* and *estP* expression profiles (*p <* 0.05, Spearman’s rank correlation), although the two genes are carried by different plasmids, i.e., p948 and p666, respectively. Surprisingly, the amplification of *cfa/Ib* failed under experimental conditions of the M-SHIME. It is unlikely that the presence of gut microbiota has created a rearrangement or mutation within the existing *cfa/Ib* gene since *cfaD* (encoding for another subunit of CFA/I adhesin, in the same operon than *cfa/Ib*) was successfully amplified and under-expressed in presence of microbiota in anaerobic stool samples [31].

Concurrently to the abiotic factors found in the human gastrointestinal tract, ETEC faces and competes with successive microbial populations for nutrients and space [36]. Here, we examined the effect of a single administration of ETEC (~ 10 log_10_) on the microbial community composition and activity in the M-SHIME. It is the first time that such modulation was followed spatially in distinct microbial gut longitudinal regions, i.e., ileum/ascending colon, and cross-sectional microenvironments, i.e. lumen/mucus, and this separates for six human-derived microbiota. We found the ileum and ascending colon to be successfully reproduced in the M-SHIME in terms of community composition, diversity, and metabolic activity [37]. Interestingly, M-SHIME was able to maintain an individual’s unique microbiota profile as donor origin was a major explanatory variable for dissimilarities in microbiota composition, together with the gut region.

Mimicking ETEC infection in M-SHIME did not result in profound shifts in the microbiome. Yet, correlations between ETEC presence and separate microbial genera from the mucosal ileum mucus and luminal colon were observed. Key changes were mostly found in the *Firmicutes* phylum with a decrease in *Clostridium butyricum*, a potentially health-promoting microbe [38]. Conversely, *Clostridium scindens* a species capable of converting primary bile acids to toxic secondary bile acids was stimulated [39]. The mucosal ileum showed blooms of taxa recognized as opportunistic pathogens: *Klebsiella variicola*, recently involved in bloodstream infections, and non-tuberculous *Mycobacterium* [40, 41]. Opportunistic pathogens are often associated with stress conditions for the host [42]. However, we demonstrated that the proliferation of opportunistic pathogens is a host-independent effect, related in this case to ETEC infection. Although limited and highly gut region-specific, the ecological features that we observed following ETEC infection may reflect an imbalance in the microbiota. A previous clinical study suggested that the increase of *Firmicutes*:*Bacteroidetes* ratios in human fecal samples 72 h post-ETEC infection resulted in a dysbiotic microbiota, even though a significant difference in the microbial diversity was not observed when comparing ETEC-free travelers to ETEC-infected travelers [9]. In our work, we did not find a significant change in bacteria diversity, which is in line with two other studies performed on fecal samples of ETEC-positive Bangladeshi patients or experimentally infected adults [10, 11].

Reflecting the microbial metabolic activity, SCFA are important determinants of interactions between the microbiota and enteric pathogens. For instance, SCFA can regulate the expression of virulence genes in EHEC and *Salmonella typhimurium* [42–44]. In our study, SCFA concentrations in the ileum and ascending colon displayed significant changes following ETEC infection mainly through the increase of the propionate concentration (ileum *p =* 0.007; ascending colon *p =* 0.01, pairwise Wilcoxon rank sum test with Holm correction). Propionate is acknowledged to be a health-promoting microbial metabolite, but direct interaction between ETEC and propionate has not yet been documented.

## Conclusions

To conclude, ETEC pathogenesis in the human gastrointestinal tract was thus far considered a “black box.” Our present study provided for the first time a detailed insight into the temporal and spatial modulation of ETEC H10407 survival, virulence, and its interactions with gut microbes by combining the two most complete and well-validated TIM-1 and M-SHIME systems worldwide. Future results will enable prospecting of preventive and/or therapeutic strategies.

## Methods

### ETEC strain and growth conditions

The prototypical ETEC strain H10407 serotype O78:H11:K80 (LT^+^, ST^+^, CFA/I^+^) isolated from a Bangladeshi patient with cholera-like syndromes was used in this study [45]. Bacteria were routinely grown under agitation (37 °C, 125 rpm, overnight) in Luria Bertani (LB) broth (BD Difco) until OD_600nm_ = 0.6 (stationary phase), to achieve a final amount of 10 log_10_ CFU in the TIM-1 inoculum.

### Inoculation and operation of the TIM-1

The experimental set-up of the TIM-1 was previously described [19]. Based on in vivo data, the TIM-1 system was programmed to simulate the physicochemical digestive conditions encountered in a healthy adult when a glass of water (main route of transmission for ETEC) is ingested (Additional file 1: Table S5) [12]. The bacterial suspension (200 mL) introduced into the TIM-1 system consists of mineral water experimentally contaminated with ETEC at a final amount of 10 log_10_ CFU. Two types of experiments were performed: gastric digestions (total duration of 60 min) and gastrointestinal digestions using the entire TIM-1 model (total duration of 300 min). Digestions were run in quadruplicate (Fig. 1a).

### TIM-1 sampling

The initial bacterial suspension (T0) was collected and samples were regularly taken during in vitro digestions from each digestive compartment (stomach, duodenum, jejunum, and ileum) (Fig. 1a). Gastric and ileal effluents were also collected on ice and pooled on 0–10, 10–20, 20–40, and 40–60 min for gastric digestions and hour-by-hour during 5 h for gastrointestinal digestions. Time 75 min represents the fraction remaining at the end of gastric digestions in the stomach and T 330 min the gastric and small intestinal residues at the end of gastrointestinal digestions. Samples collected for plating and flow cytometry were immediately treated. The number of cultivable ETEC in each digestive compartment of the TIM-1 was determined by direct plating onto LB agar (overnight incubation at 37 °C) [19]. Samples used for DNA or RNA extraction were centrifuged (6339×*g*, 10 min, 4 °C). DNA samples were stored at − 20 °C while RNA were resuspended in 500 μL RNA*later*® (Thermo Fisher Scientific) prior to storage at − 80 °C. The supernatant was stored at − 20 °C for an enterotoxin ELISA assay.

### M-SHIME experimental set-up

The M-SHIME® consists of a series of connected double-jacketed reactors (Pierreglas, Vilvoorde, Belgium). Based on in vivo data, the system reproduces the conditions of the upper and lower part of the human gastrointestinal tract, operated in a semi-continuous mode to mimic gastrointestinal transit [14, 46]. Three successive compartments simulating the stomach/combined duodenum-jejunum, the ileum, and the ascending colon were used in the set-up (Fig. 1b). The mucosal environment was reproduced in both the ileum and ascending colon compartments, incorporating microcosms (AnoxKaldnes K1 carrier, AnoxKaldnes AB) coated with type III porcine mucin-agar (Sigma-aldrich), as described by Van den Abbeele et al. [47].

### Inoculation and operation of the M-SHIME

Fresh fecal samples were collected from six healthy adults (3 women and 3 men aged from 25 to 36 years old) (Additional file 1: Table S6) in sterile airtight containers comprising an Anaerocult® A strip (Merck) to maintain anoxic conditions until processing. A 20% (w/v) fecal slurry was prepared as described by De Boever et al. [46] and inoculated in separate ascending colon vessels pre-filled with 500 mL nutritional medium (Prodigest, Zwijnaarde, Belgium) in order to obtain a final concentration of 1% (w/v) fecal material [46, 47]. All vessels were flushed with N_2_ immediately after inoculation to generate anaerobic conditions. After the initial overnight incubation of the fecal sample in the ascending colon, a semi-continuous feeding pattern with nutritional SHIME medium, simulated gastric, biliary, and pancreatic secretions was established (Additional file 1: Table S5) by pumping feed into the stomach/combined duodenum-jejunum vessel three times a day. Subsequently, this mixture was transferred to the ileum vessels, followed by the ascending colon vessels. After a transit time of 3 h in the ileum and a hydraulic residence time of 20 h in the ascending colon, the entering volume was discharged. In order to establish an ileal microbial community at low biomass concentration, a 100-μL feedback inoculation from the ascending colon vessels towards the respective ileum vessels was conducted on days 3 and 8. From day 3 onwards, the nutritional SHIME medium was supplemented with simple sugars 0.5 g L^−1^ each, i.e., glucose, fructose, galactose, maltose, and sucrose to enhance the growth of bacteria usually found in the small intestine. To mimic the lower turnover rate of the mucus environment and avoid wash-out of mucus adherent bacteria, half of the mucus beads were replaced every 2–3 days in each of the ileum and ascending colon vessels [48].

The six fecal donors were evaluated in two M-SHIME runs, each involving three individuals over the course of 20 days. A stabilization period (adaptation to the in vitro conditions) of 7 days was applied. After a pre-infection period from day 7 to 12, the system was challenged at day 13 in the SHIME ileum vessels with ETEC by inoculation of 10 log_10_ CFU, followed by a post-infectious period from day 14 to 20. Prior to the challenge, ETEC was pre-digested 2 h 30 under static conditions, to reproduce the gastro-jejunal digestion of a glass of contaminated mineral water, where physicochemical conditions were close to those found in TIM-1 (without nutritional medium, under aerobic conditions) (Additional file 1: Table S7). Low variability in microbiota between technical replicates from the same donor has been tested on a separate SHIME experiment (Additional file 1: Fig. S12).

### M-SHIME sampling

SHIME suspensions from the ileum and ascending colon vessels were sampled daily for short-chain fatty acid (SCFA) analysis. Samples were also regularly collected for DNA and RNA extractions and ELISA measurement and stored as previously explained for TIM-1 samples. Mucus samples were obtained every 2–3 days [48]. Two hundred fifty milligrams of mucus was aliquoted and stored at − 20 °C before DNA extraction.

### DNA extraction

Total DNA from the TIM-1 and M-SHIME experiments was extracted according to Geirnaert et al. [48]. The DNA samples were stored at − 20 °C and the quality was analyzed by gel electrophoresis (1.2% (w/v) agarose) (Life technologies). The DNA samples were diluted 1:10 in 1X TE buffer (Tris and EDTA) for further ETEC quantification.

### qPCR and PMA-qPCR-based quantification

Digestive samples from TIM-1 and M-SHIME were collected in duplicate and stained or not with 50 μM PMA (Interchim). The qPCR procedure was performed using the StepOnePlus Real-Time PCR system (Applied Biosystems). The total reaction volume of 10 μL contained 5 μL of Takyon Low Rox Sybr Master Mix dTTP blue (Eurogentec), 0.45 μL (10 μM) of *16S* of *Enterobacteriaceae* primers for TIM-1 and *gspD* primers for M-SHIME (Additional file 1: Table S8) [49, 50], 3.1 μL of nuclease-free water (Sigma-Aldrich), and 1 μL of template DNA. The non-template control consisted of 1 μL nuclease-free water. Each reaction was performed in triplicate in a sealed 96-well plate. The qPCR thermal cycling amplification procedure followed was previously described [19]. The melting curves of PCR amplicons were checked to ensure primer specificity and yielded a single melting peak.

ETEC pure cultures (OD_600nm_ = 0.3, exponential phase, LB broth) stained or not with PMA were used as DNA templates. Standard curves were thus generated by PCR amplification of the *16S Enterobacteriaceae* rRNA and ETEC *gspD* genes for TIM-1 and M-SHIME samples, respectively [19]. Tenfold serial dilutions of the standard were prepared in TE buffer and stored at − 20 °C until further use. As a negative control, an ETEC pure culture was subjected to lethal heat-treatment (95 °C, 15 min) and stained or not with PMA. The absence of viable ETEC in the heat-treated samples was confirmed on LB agar plates.

### Flow cytometry analysis—Live/Dead ETEC quantification

Adequate volumes of gastric or ileal effluents (Live/Dead and membrane potential kits) from TIM-1 were centrifuged (9000×*g*, 5 min, 20 °C). Pellets were resuspended into PBS at pH 7.3 to obtain approximately 6 log_10_ cells mL^−1^. Flow cytometry analysis was performed on a CyFlow SL cytometer and data were collected with FlowMax software version 2.3 (Partec) [19]. Bacteria were double-stained using the Live/Dead BacLight™ Kit (L34856 Molecular Probes) according to the protocol previously described [19].

### RNA isolation, DNAse treatment, and quality control

Total RNA was extracted from TIM-1 and SHIME digestive samples using the TRIzol® method (Invitrogen) [51]. RNA pellets were resuspended in Diethyl Pyrocarbonate (DEPC)-treated water (Invitrogen) and stored at − 80 °C. DNAse treatment with the TURBO DNA-free™ Kit (Invitrogen) was performed to remove any contaminating genomic DNA according to the manufacturer’s recommendations. The efficiency of DNAse treatment was checked by PCR [18] using e*ltB* gene-specific primers (Additional file 1: Table S8) [52]. The lack of amplification was indicative of the successful DNA removing from RNA samples. PCR products were analyzed by gel electrophoresis (1.2% (w/v) agarose). RNA was quantified using the NanoDrop ND-1000 (Thermo Fisher Scientific). A minimal concentration of 10 ng μL^−1^ was required to carry out the reverse transcription polymerase chain reaction (RT-PCR). RNA integrity was checked using the 2100 Bioanalyzer and the RNA 6000 Nano kit (Agilent Technologies) according to the manufacturer’s instructions. Only high-quality RNA was retained for subsequent transcriptional analysis.

### Quantitative reverse transcription (RT-qPCR)

RNA was reverse transcribed into complementary DNA (cDNA) by using the PrimeScript™ RT Reagent Kit (TAKARA Bio Inc) according to the manufacturer’s instructions. qPCR was performed on the cDNA. All the primers used at a concentration of 300 nM (except *tia* and *leoA* primers at 100 nM) are listed in Additional file 1: Table S8 [49, 50, 52–58]. qPCR data were analyzed using the comparative E^−ΔΔCt^ method and normalized with the reference genes *arcA* and *gapA*, after controlling their stability using BestKeeper software. The amplification efficiency of each reference gene was determined by the generation of a standard curve based on a tenfold dilution series of a set of cDNA samples from each compartment of the TIM-1 and SHIME models. The amplification efficiency was calculated from the slope of the standard curves E = 10^(− 1/slope)^; *E* values between 90 and 110% were considered acceptable. Differences in the relative expression levels of each virulence gene were calculated as follows: ΔΔCt = (Ct_target gene_ – Ct_reference gene_)_at time *t*_ – (Ct_target gene_ – Ct_reference gene_)_at time t0_, and data were derived from E^-ΔΔCt^. T0 represents the initial time.

### LT-monosialoganglioside (GM1) ELISA

LT enterotoxins were measured in supernatants collected from the TIM-1 and M-SHIME as previously described [59]. Optical density was read at 450 nm using the multiscan Tecan Infinite® 200 PRO. LT toxin concentrations were expressed in pg mL^−1^.

### SCFA production

Luminal samples from the M-SHIME were diluted 1:2 with miliQ water to a total volume of 2 mL. SCFA production was measured using capillary gas chromatography coupled to a flame ionization detector after diethyl ether extraction [60]. SCFA concentrations were expressed in mM.

### Statistical analysis of ETEC data

All statistical analyses were performed in R studio, version 3.5.1. All formal hypothesis tests were conducted on the 5% significance level (*p ≤* 0.05). The assumptions of normality and heterogeneity of variances were verified based on visual inspection of QQ-plots and Shapiro-Wilk (stats4_3.4.2) and Levene’s test (car_2.1-5), preceding statistical hypothesis testing to assess pairwise comparison of (i) ETEC survival at time *T* in comparison with the transit marker of the TIM-1 system, (ii) ETEC survival at time *T* in comparison with the initial inoculum (T0) in the M-SHIME, (iii) ETEC survival between luminal and mucosal microenvironments at time *T*, (iv) the log_2_ fold change in gene expression at time *T* in comparison with the T0, and (v) the LT enterotoxin production at time *T* in comparison with T0. The assumptions were not met for most variables, in which case a non-parametric Mann-Whitney (Wilcoxon rank sum) test with Holm correction was performed (for i, ii, iii hypotheses), and a Nemenyi post hoc test was conducted following a significant Friedman test (for iv and v hypotheses), using the PMCMR package. Statistical hypothesis testing to assess the effect of ETEC infection on the metabolic activity (SCFA) was performed by using the Kruskal-Wallis rank sum test, followed by pairwise Wilcoxon rank sum tests with Holm correction for multiple testing.

### Microbial community analysis

Next-generation *16S* rRNA gene amplicon sequencing of the V3-V4 region (341F-785R) was performed by LGC Genomics (Teddington), on an Illumina MiSeq platform using Illumina V3 chemistry (Illumina), as previously described [60]. The sequence data have been submitted to the NCBI (National Center for Biotechnology Information) database under accession number PRJNA562529 [61].

### Bioinformatics analysis of amplicon data

The mothur software package (v.1.40.5) and guidelines were used to process the Illumina amplicon sequencing data generated by LGC Genomics [62]. OTUs were defined as a collection of sequences with a length between 400 and 428 nucleotides that are found to be more than 97% similar to one another in the V3–V4 region of their *16S* rRNA gene after OptiClust clustering [63–66]. Taxonomy was assigned using the RDP database [67, 68]. The shared file, containing the number of reads observed for each OTU in each sample, was loaded into R version 3.5.1 (2018-09-04) (R Core Team, 2016). Singletons were removed [67]. For the most abundant OTUs, the sequences retrieved from the 3% dissimilarity level fasta file, obtained in mothur, were classified through the RDP web interface using the RDP SeqMatch tool (restricting the search to type strains with only near-full-length good quality sequences) and blasted in NCBI (National Center for Biotechnology Information) against the *16S* rRNA gene sequences, selecting only type material, with optimization of the BLAST algorithm for highly similar sequences (accession date: September 2018) [68–70]. Although a level of uncertainty is introduced by classification to the species level based on short 300 bp reads, the best hit returned by both databases is used to refer to interesting OTUs in the “Results” section of this article. In case of inconsistencies between the RDP SeqMatch tool and NCBI BLAST, no species-level classification was mentioned. A more detailed overview of the RDP Seqmatch and NCBI BLAST results for the most abundant and significant OTUs (best hit as well as the next best hits) can be found in Additional file 1: Table S9.

### Statistical analysis of amplicon and metabolic data

All statistical analyses were performed in R, version 3.5.1 (2018–09-04) (R Core Team, 2016). All formal hypothesis tests were conducted on the 5% significance level. To visualize differences in microbial community composition between donors, period (e.g., pre- and post-infection), and gut region (e.g., ileum lumen, ileum mucus, ascending lumen, and ascending mucus), ordination and clustering techniques were applied. For these purposes, the shared file was further processed to remove OTUs with too low abundance according to the arbitrary cutoffs described by McMurdie and Holmes [69]. An OTU should be observed in 5% of the samples and read counts should exceed 0.5 times the number of samples [69]. Rarefaction curves were constructed to assure that the samples were sequenced in sufficient depth [71]. To deal with differences in sampling depth, proportional data transformed on the common scale to the lowest number of reads was used for the purpose of visualization [65]. Principle coordinate analysis (PCoA; package stats4_3.3.1) [72, 73] was conducted based on the abundance-based Jaccard dissimilarity matrix (package vegan_2.4–0) [71, 74, 75] and visualized with ggplot2_2.1.0. This procedure was repeated on species and genus levels focusing on the comparison between gut regions, donor samples, and the comparison between periods. In both gut regions, the pre-infection was defined from day 7 to 12, and the post-infection from day 13 to 20. On the genus level, weighed averages of genera abundances were a posteriori added to the ordination plot using the wascores function in vegan [71]. To confirm the trends, observed data was clustered by means of an Unweighted Pair-Grouped Method using arithmetic Averages (UPGMA) clustering method (cluster_2.0.4) [76]. The significance of the observed group separation between gut region, donor, and period in the PCoA was assessed with a Permutational Multivariate Analysis of Variance (PERMANOVA) using distance matrixes (package vegan_2.4–0) [71, 73]. Prior to this formal hypothesis testing, the assumption of similar multivariate dispersions was evaluated.

When comparing the effects, the influence of the gut region, donor, and period was determined by applying a distance-based redundancy analysis (db RDA) using the abundance-based Jaccard distance as a response variable (vegan) [71, 73]. The factor donor is used as a constraint with the effect of the gut region or period being partialled out. Interpretation of the results is preceded by a permutation test of the RDA results to confirm that a linear relationship exists between the response data and the exploratory variables. The constrained fraction of the variance, explained by the exploratory variables, is adjusted by applying Ezekiel’s formula [75].

In order to find statistically significant differences in species abundance between the pre- and post-infection periods, the DESeq package was applied [67, 77]. The factors period, gut region, and donor were used in the design formula. Statistical differences between the pre- and post-infection were determined using a Wilcoxon signed rank test on the proportional data. The evolution of the microbial community structure throughout the M-SHIME run was followed up by computing the richness (Chao 1) and Simpson diversity index on the DESeq normalized, unfiltered data (package vegan_2.4–4).

## Supplementary information

**Additional file 1: Fig. S1.** [Global ETEC survival percentages in the cumulated ileal effluents of the TIM-1]. **Fig. S2.** [Live/Dead flow cytometry for accurate determination of ETEC membrane physiology in the TIM-1 gastric and ileal effluents]. **Fig. S3.** [Spearman correlation between virulence gene expression, LT toxin production and/or ETEC survival in the TIM-1 and M-SHIME]. **Fig. S4.** [Phylum and species levels microbial community composition of the luminal gut regions]. **Fig. S5.** [Phylum and species levels microbial community composition of the mucosal gut regions]. **Fig. S6.** [Gut microbes concentration in the ileum and ascending colon lumen determined by total flora *16S* rRNA gene quantification]. **Fig. S7.** [Simpson alpha diversity index over time for the six donors according to the luminal (L) and mucosal (M) gut regions]. **Fig. S8.** [db-RDA triplots showing the relationship of A) gut regions, B) donors and C) period pre- *vs* post-infection as explanatory variables to the microbial community structure at genus level]. **Fig. S9.** [db-RDA triplots showing the effect of ETEC pre- and post-infection on distinct microbial genera according to the gut regions]. **Fig. S10.** [SCFA concentrations (mM) in the ileum (ILE) and ascending colon (ASC) compartments of the M-SHIME]. **Fig. S11.** [Spearman correlations between the main SCFA produced in the ascending colon and ETEC survival, LT toxin production, and expression of virulence genes encoding for the enterotoxins]. **Fig. S12.** [High reproducibility in SCFA concentrations and microbiota composition between replicates from a same donor in a separate SHIME experiment]. **Table S1.** [ETEC intracellular pH (pHi) in the TIM-1 system]. **Table S2.** [ETEC membrane potential in the TIM-1 system]. **Table S3.** [Log_2_ fold changes in virulence genes expression in the TIM-1]. **Table S4.** [Log_2_ fold changes in virulence genes expression in the M-SHIME]. **Table S5.** [Parameters of the TIM-1 and M-SHIME systems]. **Table S6.** [General characteristic of the fecal donors involved in the M-SHIME experiments]. **Table S7.** [Static in vitro gastro-jejunal digestion procedure]. **Table S8.** [ETEC primers used in the study]. **Table S9.** [RDP Seqmatch and NCBI BLAST results for the most abundant species and/or species of interest in the M-SHIME].

## Data Availability

The raw data of 16S rRNA gene libraries generated during this study is publicly available at the Sequence Read Archive (SRA) portal of NCBI under accession number PRJNA562529 [61]. The other data generated or analyzed during this study are included in this published article and its supplementary information filesThe other data generated or analyzed during.
