# Supplementary material for: Spatial and temporal modulation of enterotoxigenic E. coli H10407 pathogenesis and interplay with microbiota in human gut models
Source: BMC Biol. 2020 Oct 14;18:141. doi: 10.1186/s12915-020-00860-x (PMC7559199; doi:10.1186/s12915-020-00860-x)
Supplement: Supplementary file 1 — Additional file 1: Fig. S1. [Global ETEC survival percentages in the cumulated ileal effluents of the TIM-1]. Fig. S2. [Live/Dead flow cytometry for accurate determination of ETEC membrane physiology in the TIM-1 gastric and ileal effluents]. Fig. S3. [Spearman correlation between virulence gene expression, LT toxin production and/or ETEC survival in the TIM-1 and M-SHIME]. Fig. S4. [Phylum and species levels microbial community composition of the luminal gut regions]. Fig. S5. [Phylum and species levels microbial community composition of the mucosal gut regions]. Fig. S6. [Gut microbes concentration in the ileum and ascending colon lumen determined by total flora 16S rRNA gene quantification]. Fig. S7. [Simpson alpha diversity index over time for the six donors according to the luminal (L) and mucosal (M) gut regions]. Fig. S8. [db-RDA triplots showing the relationship of A) gut regions, B) donors and C) period pre- vs post-infection as explanatory variables to the microbial community structure at genus level]. Fig. S9. [db-RDA triplots showing the effect of ETEC pre- and post-infection on distinct microbial genera according to the gut regions]. Fig. S10. [SCFA concentrations (mM) in the ileum (ILE) and ascending colon (ASC) compartments of the M-SHIME]. Fig. S11. [Spearman correlations between the main SCFA produced in the ascending colon and ETEC survival, LT toxin production, and expression of virulence genes encoding for the enterotoxins]. Fig. S12. [High reproducibility in SCFA concentrations and microbiota composition between replicates from a same donor in a separate SHIME experiment]. Table S1. [ETEC intracellular pH (pHi) in the TIM-1 system]. Table S2. [ETEC membrane potential in the TIM-1 system]. Table S3. [Log2 fold changes in virulence genes expression in the TIM-1]. Table S4. [Log2 fold changes in virulence genes expression in the M-SHIME]. Table S5. [Parameters of the TIM-1 and M-SHIME systems]. Table S6. [General characteristic of the fecal donors [file 12915_2020_860_MOESM1_ESM.docx]

Supplementary Information for

**Spatial and temporal modulation of enterotoxigenic *E. coli* H10407 pathogenesis and interplay with microbiota in human gut models**

Charlène Roussel^1,2^, Kim De Paepe^2^, Wessam Galia^3^, Jana De Bodt^2^, Sandrine Chalancon^1^, Françoise Leriche^4^, Nathalie Ballet^5^, Sylvain Denis^1^, Monique Alric^1^, Tom Van de Wiele^2¶*^, Stéphanie Blanquet-Diot^1¶*^

^1^ Université Clermont Auvergne, UMR UCA-INRA 454 MEDIS, Microbiology Digestive Environment and Health, Clermont-Ferrand, France

^2^ CMET, Center for Microbial Ecology and Technology, Department of Biotechnology, Faculty of Bioscience Engineering, Ghent University, Belgium

^3^ UMR 5557 Microbial Ecology, Research group on bacterial opportunistic pathogens and environment, CNRS, VetAgro Sup, Lyon, France

^4^ Unité de recherche Fromagère, VetAgro Sup, Lempdes, France

^5^ Lesaffre International, Lesaffre group, Marcq-en-Baroeul, France

**^*^** Corresponding authors

E-mail: [stephanie.blanquet@uca.fr](mailto:stephanie.blanquet@uca.fr) (SBD)

E-mail: [Tom.VandeWiele@UGent.be](file:///C:\Users\depae\Dropbox\EM\review2\Tom.VandeWiele@UGent.be) (TVW)

^¶^These authors contributed equally to this work.

**This PDF Additional File 1 includes:**

Figures S1 to S11

Tables S1 to S8

Supplementary text


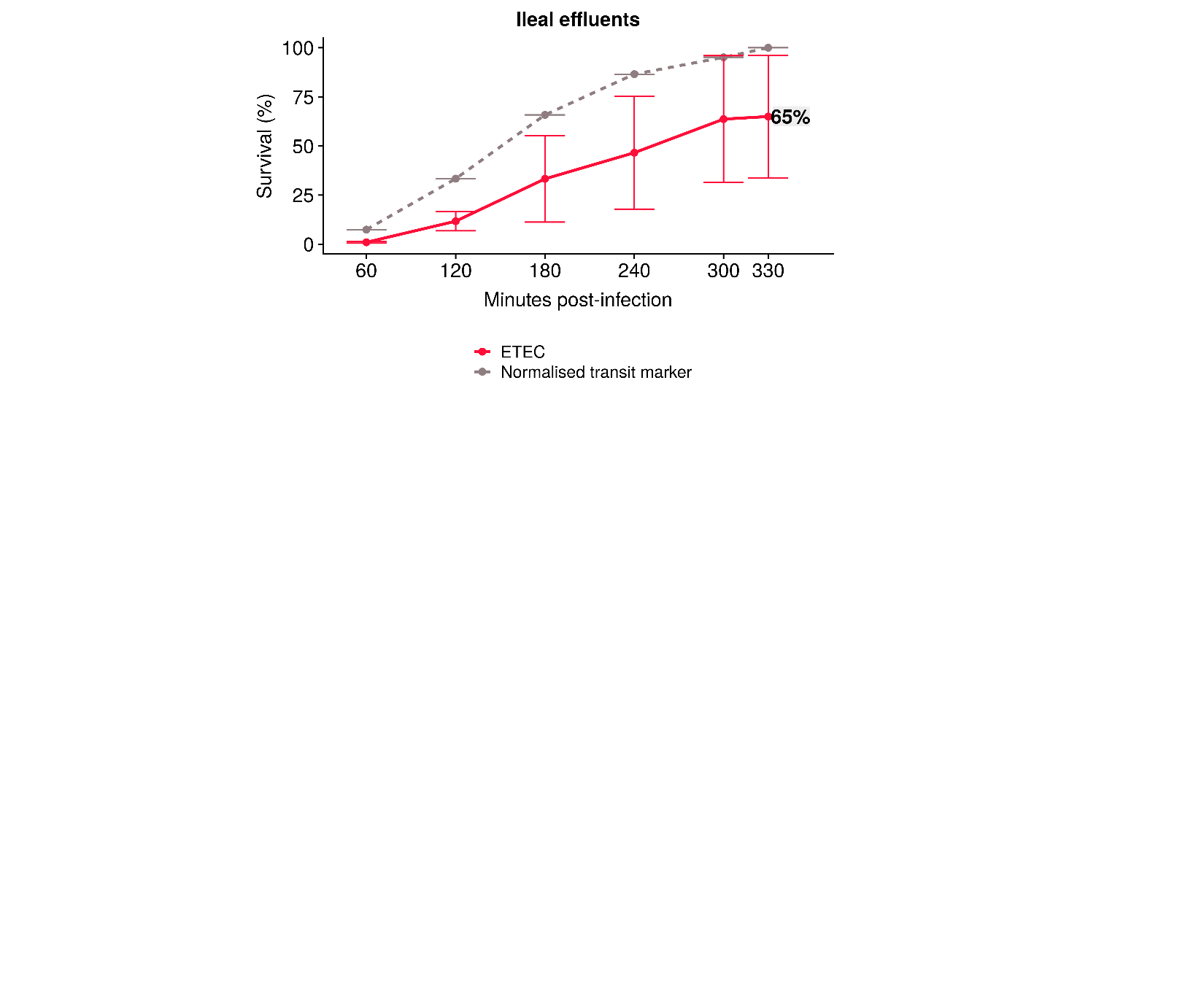


**Fig. S1. Global ETEC survival percentages in the cumulated ileal effluents of the TIM-1.** Results are expressed as mean percentages ETEC survival ± SD of four independent replicates (red line), compared with an inert and non-absorbable transit marker indicating 100% survival (grey dashed line). The global ETEC survival rate at the end of is calculated as followed: $Global survival rate (\%)=\frac{number of ETEC cells\left( T1+T2+T3+T4+T5+end digestion residues \right)x100}{number of ETEC cells T0}$. No statistical significant differences were found between ETEC and transit marker kinetics, as determined by Pairwise Wilcoxon Rank Sum Tests with Holm correction.

**
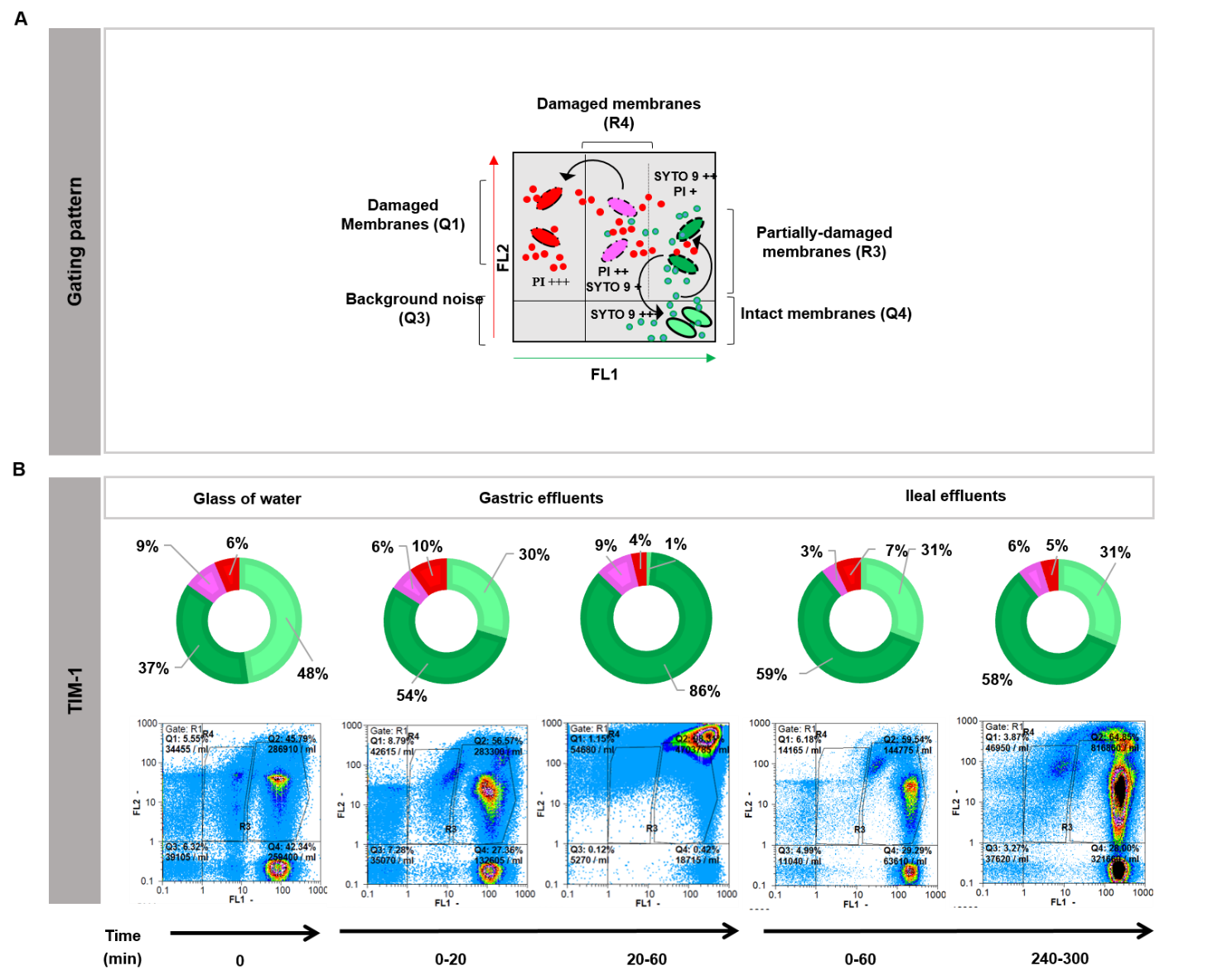
**

**Fig. S2. Live/Dead flow cytometry for accurate determination of ETEC membrane physiology in the TIM-1 gastric and ileal effluents.** A) Schematic representation of four ETEC subpopulations discriminated on the cytogram after SYTO9 and PI staining, according to the green (FL1) and red (FL2) fluorescence signals. B) The doughnut charts represented the average percentages of ETEC cell states from two independent replicates over time. Cytograms are shown for only one replicate.


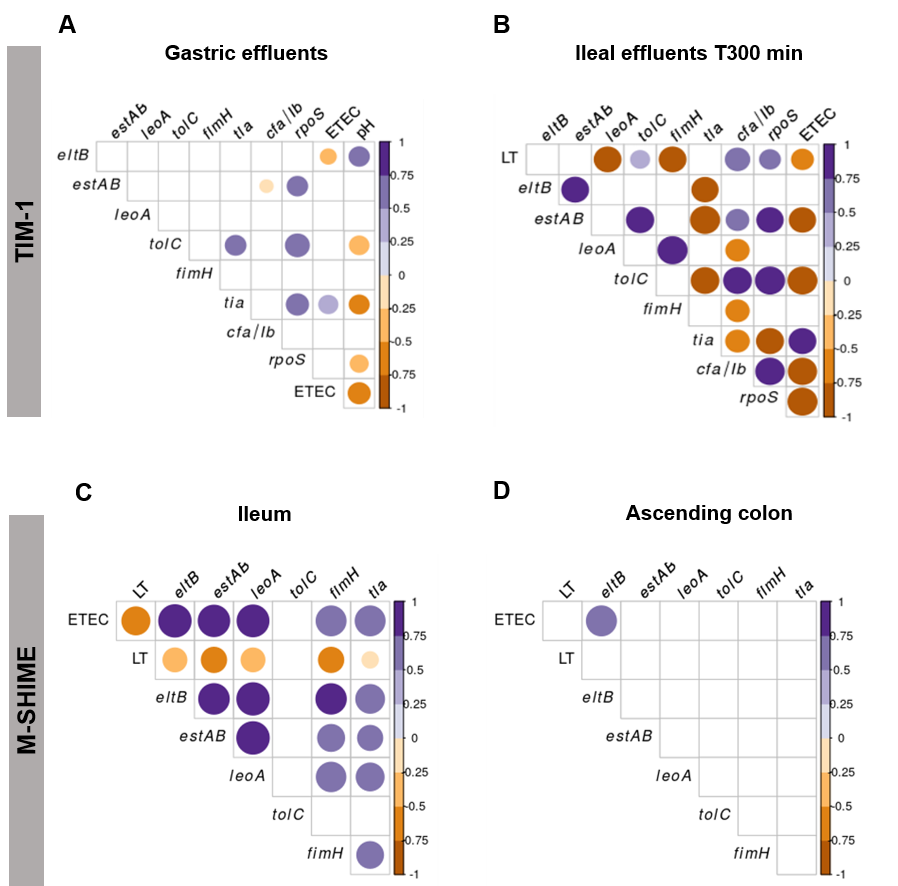
Fig. S3. Spearman correlation between virulence gene expression, LT toxin production and/or ETEC survival in the TIM-1 and M-SHIME. The size and color of the circles correspond to the magnitude and sign of correlations. Only significant correlations are shown (*p*< 0.05) in A) TIM-1 gastric (20-60 min; n=4) and B) ileal effluents (300 min; n=4) and in M-SHIME C) ileum (pool of time 1, 3, and 27 h; n=6) D) ascending colon (pool of time 5, 20 and 29 h; n=6).

**
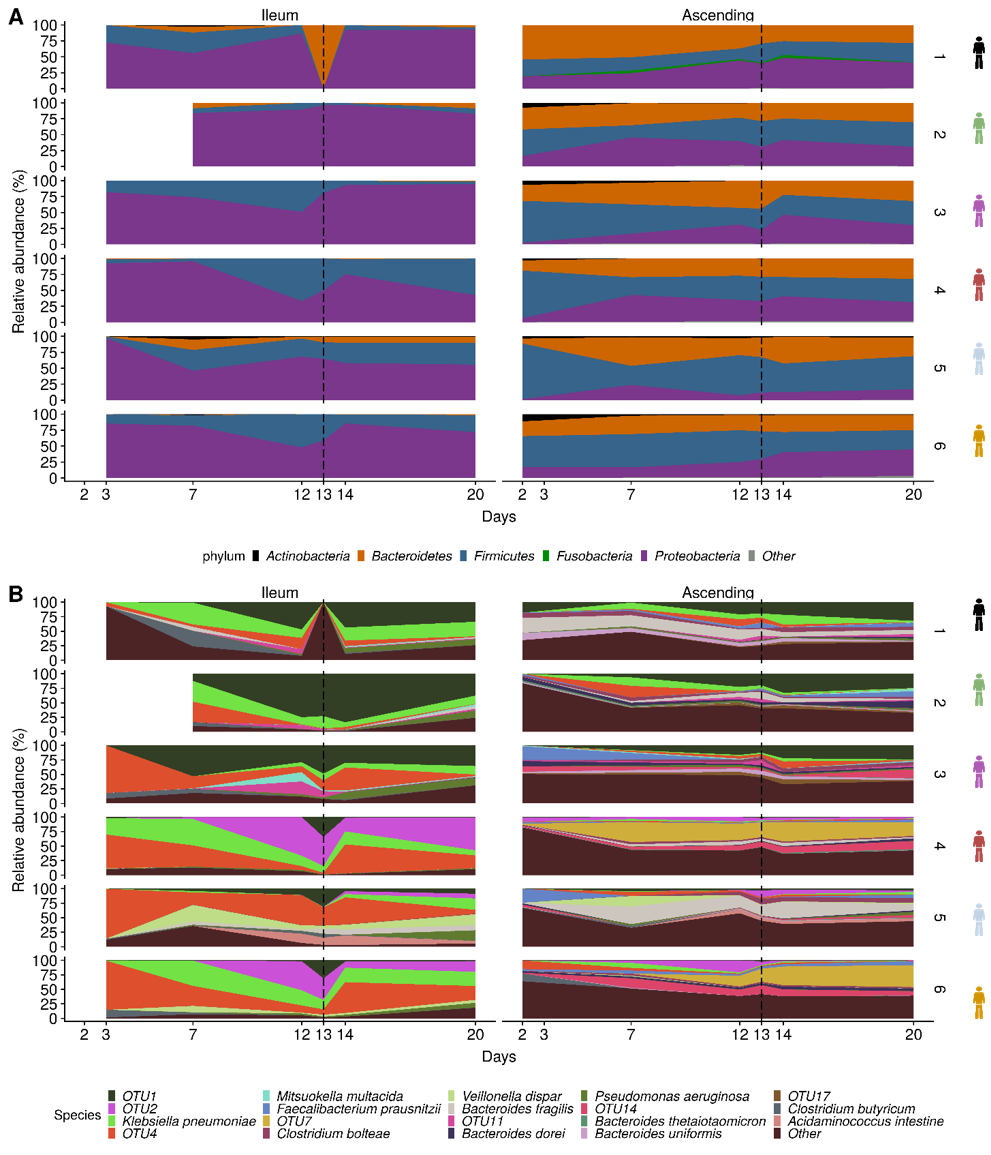
Fig. S4. Phylum and species levels microbial community composition of the luminal gut regions.** The area graphs show the relative abundance of the A) 5 most abundant phyla, and B) 19 most abundant species in the luminal ileum and ascending colon from six different donors over the course of 20 days fermentation, as determined by amplicon sequencing. ETEC infection is demarcated by the dashed line at day 13. Species were annotated using the RDP SeqMatch and NCBI BLAST. For species that could not unambiguously be identified at species level, the OTU identifier is displayed.


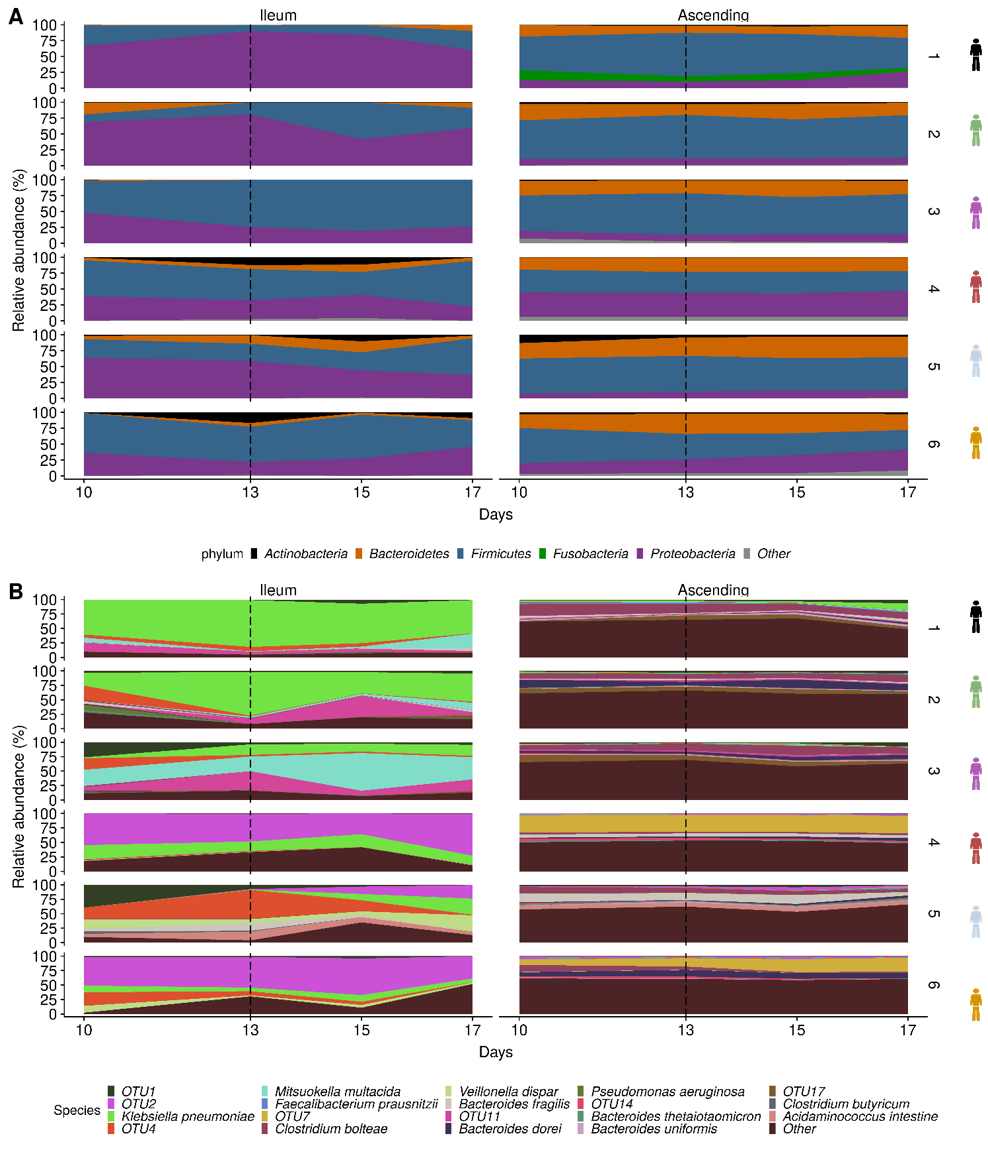
Fig. S5. Phylum and species levels microbial community composition of the mucosal gut regions. The area graphs show the relative abundance of the A) 5 most abundant phyla, and B) 19 most abundant species in the mucosal ileum and ascending colon from six different donors over the course of 20 days fermentation, as determined by amplicon sequencing. ETEC infection is demarcated by the dashed line at day 13. Species were annotated using the RDP SeqMatch and NCBI BLAST. For species that could not unambiguously be identified at species level, the OTU identifier is displayed.


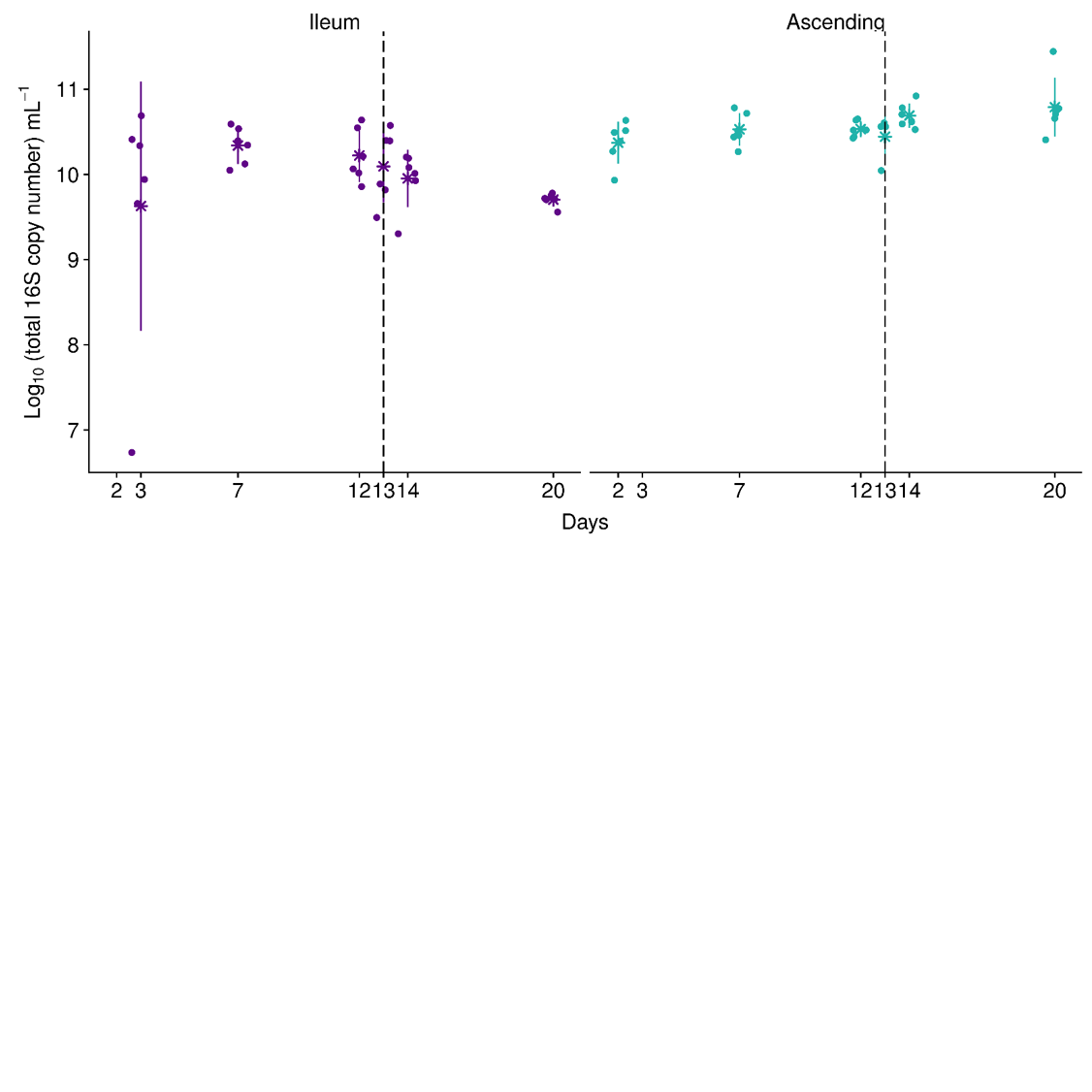
Fig. S6. Gut microbes concentration in the ileum and ascending colon lumen determined by total flora *16S* rRNA gene quantification. Total bacterial *16S* rRNA gene copy number was quantified by qPCR with 338F and 518R total flora primers amplifying the V3 region, synthesized by Biolegio (Nijmegen, The Netherlands) [39]. The qPCR procedure was performed as described by Geirnaert et al., 2015 [26]. A standard was generated by cloning the 63F-1378R amplicon obtained from *Bifidobacterium breve* JCM 7019 into a pCR2.1®-TOPO®TA vector in *E. coli* by means of the TOPO®TA kit for subcloning (Promega, Madison, WI, US) (Lane, 1991). Plasmid DNA was extracted with the PureYield*TM* Plasmid Miniprep kit (Promega, Madison, WI, US) and the concentration was measured with a NanoDrop spectrophotometer (Thermo Fisher Scientific, San José, US) and calculated back to the *16S* rRNA gene copy number, which amounted 2x10^10^ copies *μ*L^−1^. A tenfold dilution series of the standard (10^3^ to 10^9^) was analyzed in a preliminary test and the quantification limit was set at 10^3^ copies *μ*L^−1^.

**
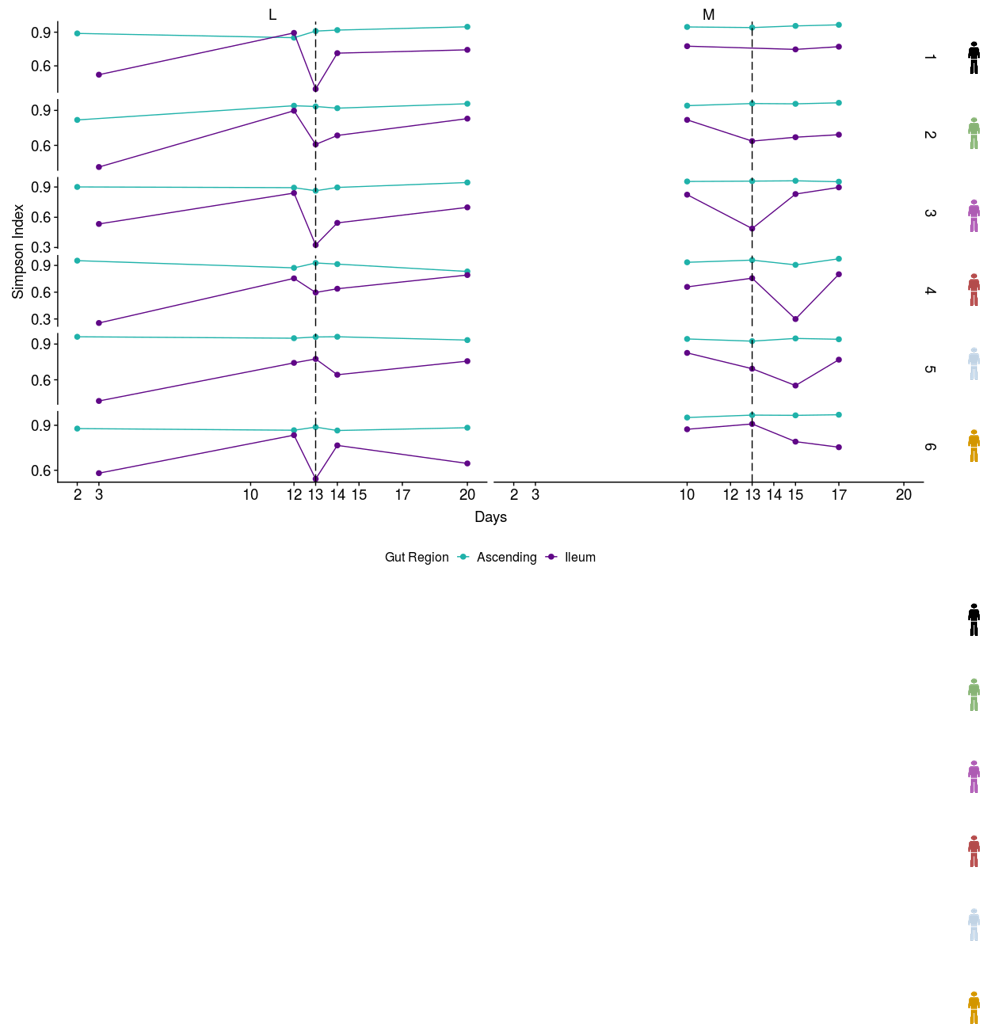
Fig. S7. Simpson alpha diversity index over time for the six donors according to the luminal (L) and mucosal (M) gut regions.** Simpson's Diversity Index is a measure of diversity which takes into account the number of species present, as well as the relative abundance of each species. It gives more weight to common or dominant species, as determined by amplicon sequencing. The diversity index ranges between 0 (no diversity) and 1 (infinite diversity). ETEC infection is demarcated by the dashed line at day 13.

**
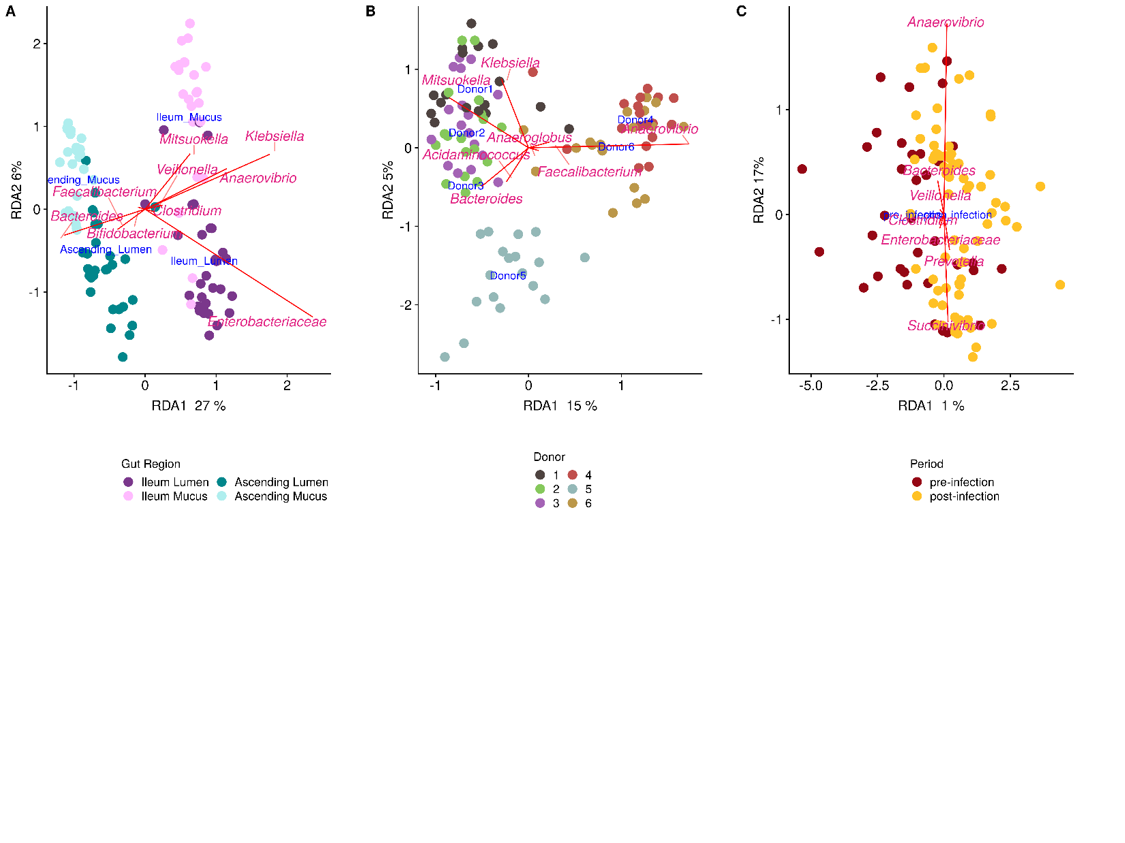
**

**Fig. S8. db-RDA triplots showing the relationship of A) gut regions, B) donors and C) period pre- *vs* post-infection as explanatory variables to the microbial community structure at genus level.** Factor levels are represented as centroids in blue. Genus scores in pink show the direction of higher abundance of a particular genus. Factors contributed to the variation of the microbial community structure at A) 19.5% (*p=* 0.001); B) 6.5% (*p=* 0.001) and C) 0.9% (*p=* 0.53).

**
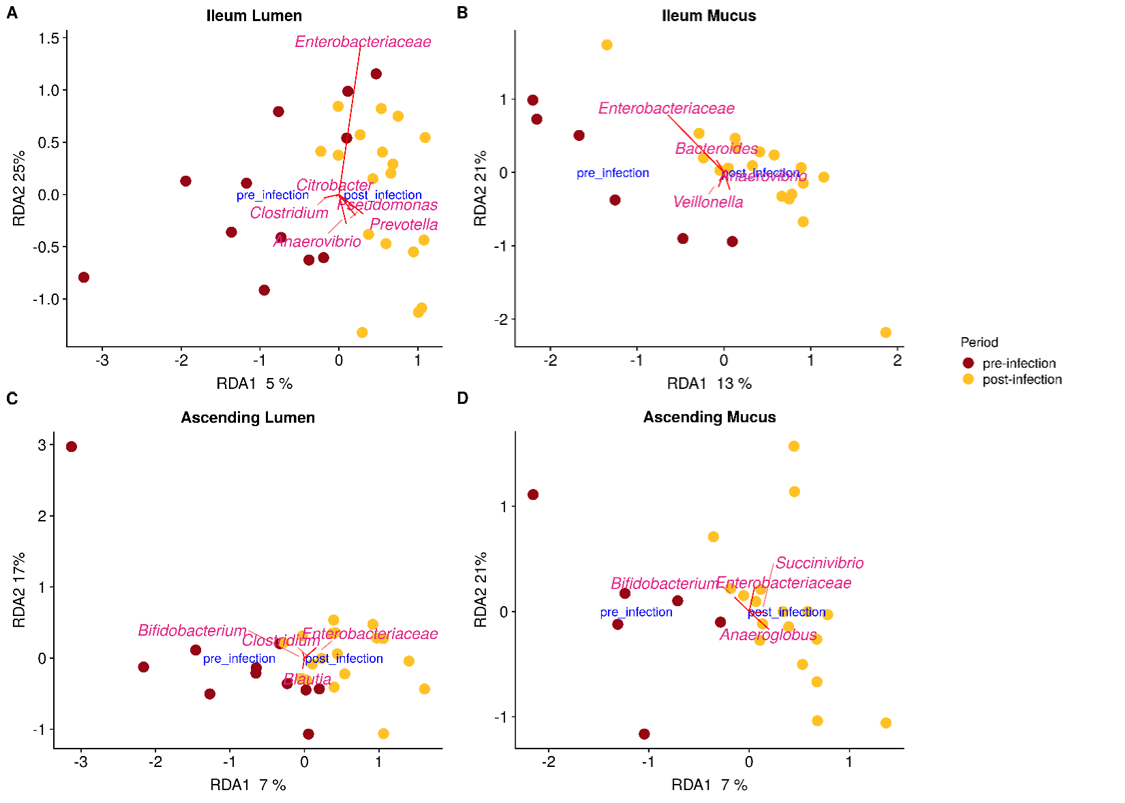
**

Fig. S9. db-RDA triplots showing the effect of ETEC pre- and post-infection on distinct microbial genera according to the gut regions. Factor levels are represented as centroids in blue. Genus scores in pink show the direction of higher abundance of a particular genus. The period contributed to the variation of the microbial community structure in A) Ileum Lumen 1.3% (*p=* 0.21); B) Ileum Mucus 2.6% (*p=* 0.01); C) Ascending Lumen 1.6% (*p=* 0.04) and D) Ascending Mucus 1.2% (*p=* 0.23).


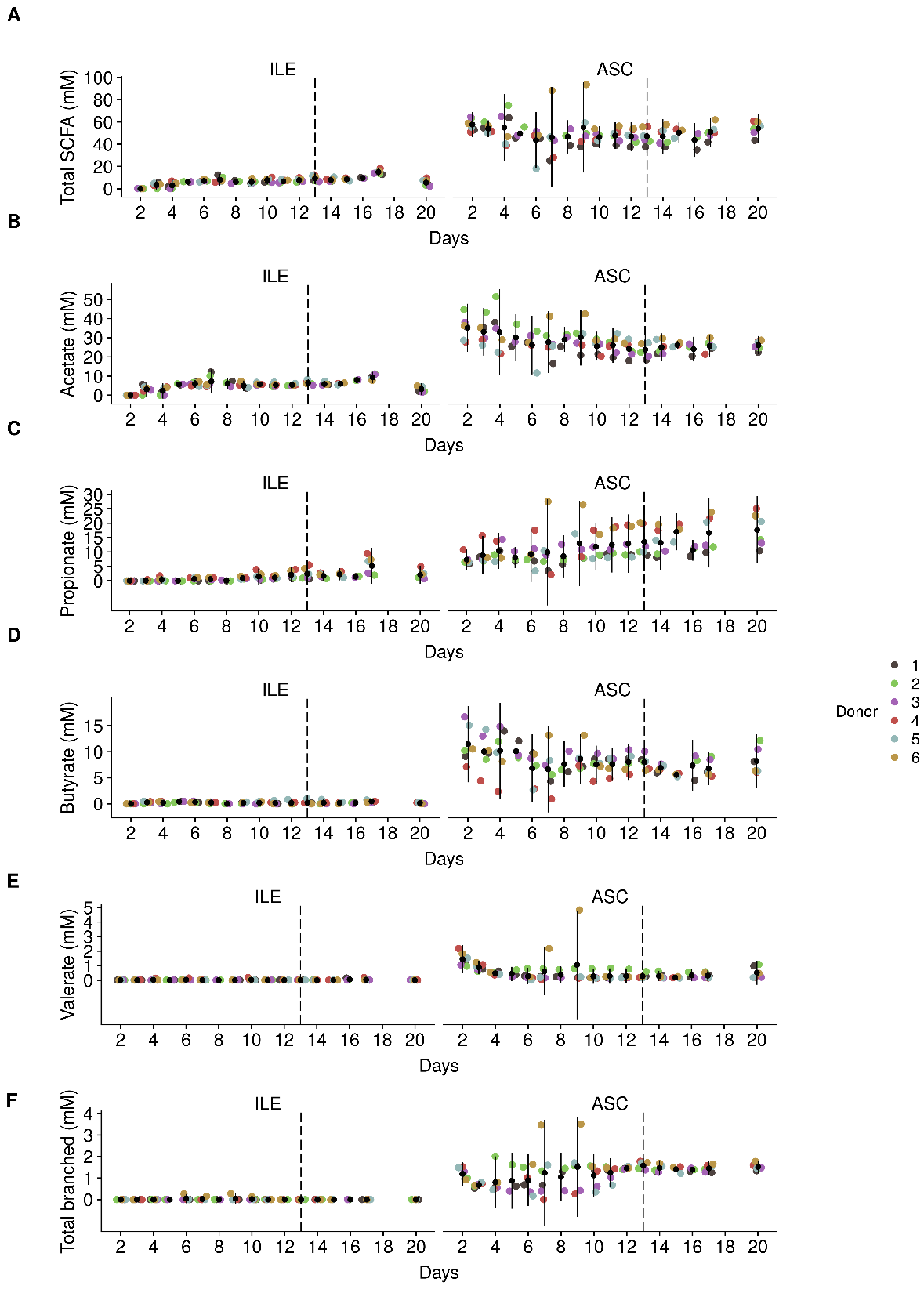
**Fig. S10. SCFA concentrations (mM) in the ileum (ILE) and ascending colon (ASC) compartments of the M-SHIME.** Metabolic microbial activity from six different donors in the course of 20 days fermentation. ETEC infection is demarcated by the dashed line at day 13.


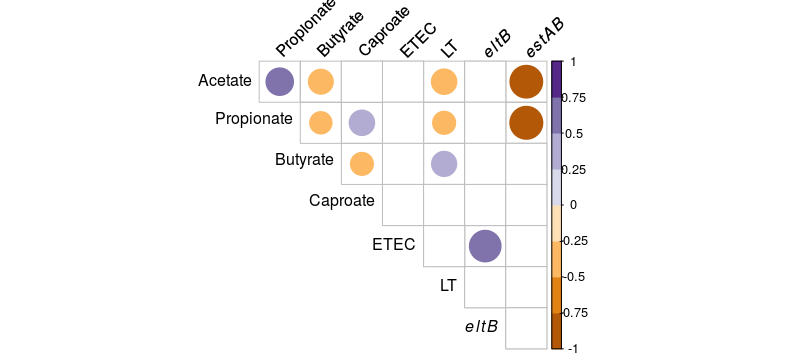


**Fig. S11. Spearman correlations between the main SCFA produced in the ascending colon and ETEC survival, LT toxin production, and expression of virulence genes encoding for the enterotoxins.** Correlations are made only for the post-infection period. Significant negative and/or positive correlations are denoted by the circles (*p*< 0.05).


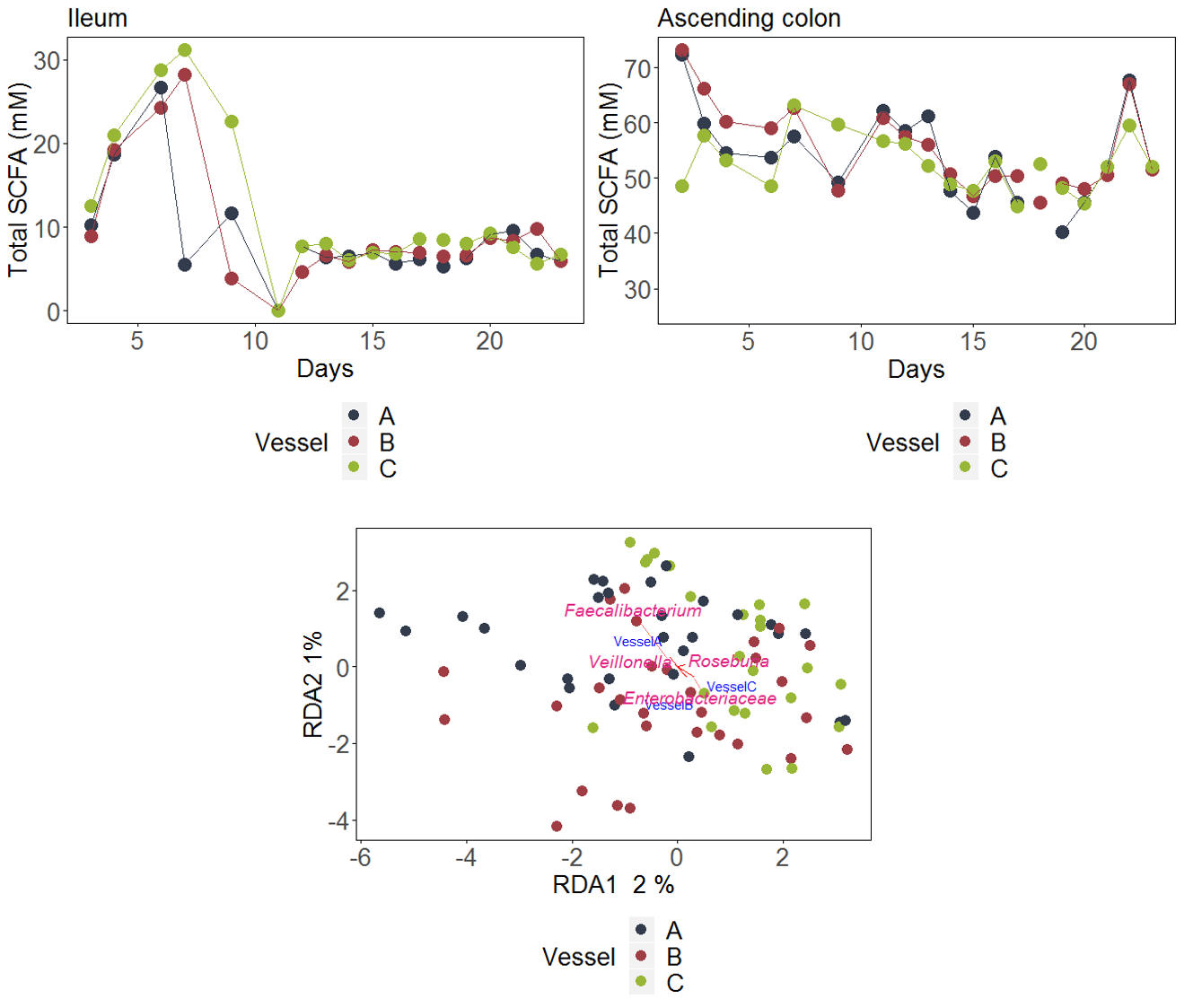


**Fig. S12. High reproducibility in SCFA concentrations and microbiota composition between replicates from a same donor in a separate SHIME experiment.** Metabolic microbial activity from one donor in triplicate bioreactors (vessel A, B, C) in the course of 22 days fermentation is shown in the two graphs on top according to the gut region. No statistical difference was found between replicates. At metagenomic level (graph down), the db-RDA shows no significant variation in the microbial composition at genus level for the three vessels. The variability between the three vessels explained only 2.54% (*p=*0.501) of the overall microbial change.

**Table S1. ETEC intracellular pH (pHi) in the TIM-1 system.** The carboxyfluorescein diacetate succinimidyl ester CFDA-SE Vybrant Kit (CFDA SE cell Tracer, Kit V12883 Thermo Fisher Scientific, Waltham, USA) was used to measure ETEC pHi. Bacterial pellets were resuspended in McIlvaine buffer at pH 7.3 (citric acid: 0.1M; disodium hydrogenophosphate: 0.2M), supplemented with 1 mM EDTA. Samples were incubated with CFDA-SE (5 µL mL^-1^; 8.9 mM in DMSO) 10 min, 40°C under slight agitation in the dark and centrifuged 2 min 10,000*×g* at room temperature. The remaining pellets were resuspended in McIlvaine’s buffer without EDTA at the pH value equal to the pH of the sample (gastric or ileal samples) before flow cytometry analysis. The fluorescence intensity of the cells was directly linked to the pHi in the range of the calibration curves. Calibration curves were determined for each cell sample. After staining, cells were centrifuged (10,000 *×g*, 2 min, at room temperature) and resuspended in McIlvaine's buffer with pH values ranging from 3 to 8. Five microliters of nigericin (Molecular Probes, Waltham, USA) (0.2 mM in DMSO) and 5 μL of valinomycin (Sigma, Saint-Quentin-Fallavier, France) (0.2 mM in DMSO) were added to equilibrate the pHi and the external pH (pHext) and kept at room temperature for 2 min before flow cytometry analysis. The fluorescence intensity from each sample (e.g. initial inoculum, T 20 in the stomach, T 180 in the duodenum and T 300 in the ileum) was obtained from the calibration curves expressed according to the pHi. Extracellular pH (pHext) are also given in each digestive compartment. The table shows the mean of two independent replicates ± SD.

| Digestive compartment | pHext | pHi |
| --- | --- | --- |
| Inoculum (T0) | 6.5 | 6.6 ± 0.1 |
| Stomach (T20 min) | 2.5 ± 0.2 | 3.0 ± 0.1 |
| Duodenum (T180 min) | 6.5 ± 0.2 | 5.8 ± 0.3 |
| Ileum (T300 min) | 7.0 ± 0.1 | 6.1 ± 0.2 |

**Table S2. ETEC membrane potential in the TIM-1 system.** The membrane potential probe 3,3’-diethyloxacarbocyanine iodide DiOC_2_(3) Kit (BacLight^TM^ Kit B34950 Thermo Fisher Scientific, Waltham, USA) and the proton ionophore carbonyl cyanide m-chlorophenyl hydrazone (CCCP) supplied by the kit were used according to the manufacturer’s instructions. Briefly, DiOC_2_(3) at low concentration exhibits green fluorescence in all bacterial cells. However, the dye becomes more concentrated in healthy cells that are maintaining a membrane potential, causing the dye to self-associate and the fluorescence emission to shift to red. The CCCP was used as a control to eradicate the proton gradient, eliminating thus the bacterial membrane potential (depolarized membranes). Analysis was performed using fluorescence emission ratio detection for bacteria incubated with 30 µM DiOC_2_(3) for 30 minutes at room temperature in the dark in either the presence or absence of 5 µM CCCP. According to the fluorescence intensity (FI) ratio (sample/control), membranes are considered to be depolarized when FI ratio is approaching 1 and polarized when exceeding 1.2. The table shows the mean of two independent replicates ± SD.

| Digestive compartment | Time point (min) | FI ratio sample/control |
| --- | --- | --- |
| Inoculum | 0 | 1.41 ± 0.35 |
| Gastric effluents | 20 | 1.42 ± 0.68 |
|  | 60 | 1.17 ± 0.38 |
| Ileal effluents | 60 | 1.51 ± 1.23 |
|  | 120 | 2.12 ± 0.39 |
|  | 180 | 1.50 ± 0.09 |
|  | 240 | 1.46 ± 0.26 |
|  | 300 | 1.31 ± 0.17 |

**Table S3. Log_2_ fold changes in virulence genes expression in the TIM-1.** STO: gastric effluents; ILE: ileal effluents.

| **Replicate** | **Minutes post-infection** | **Gut Region** | ***eltB*** | ***estP*** | ***leoA*** | ***tolC*** | ***fimH*** | ***tia*** | ***cfa/Ib*** | ***rpoS*** |
| --- | --- | --- | --- | --- | --- | --- | --- | --- | --- | --- |
| \| 1 \| \| --- \| \| 1 \| \| 1 \| \| 2 \| \| 2 \| \| 2 \| \| 3 \| \| 3 \| \| 3 \| \| 4 \| \| 4 \| \| 4 \| \| 1 \| \| 1 \| \| 1 \| \| 1 \| \| 1 \| \| 2 \| \| 2 \| \| 2 \| \| 2 \| \| 2 \| \| 3 \| \| 3 \| \| 3 \| \| 3 \| \| 3 \| \| 4 \| \| 4 \| \| 4 \| \| 4 \| \| 4 \| | \| 10 \| \| --- \| \| 20 \| \| 60 \| \| 10 \| \| 20 \| \| 60 \| \| 10 \| \| 20 \| \| 60 \| \| 10 \| \| 20 \| \| 60 \| \| 60 \| \| 120 \| \| 180 \| \| 240 \| \| 300 \| \| 60 \| \| 120 \| \| 180 \| \| 240 \| \| 300 \| \| 60 \| \| 120 \| \| 180 \| \| 240 \| \| 300 \| \| 60 \| \| 120 \| \| 180 \| \| 240 \| \| 300 \| | \| STO \| \| --- \| \| STO \| \| STO \| \| STO \| \| STO \| \| STO \| \| STO \| \| STO \| \| STO \| \| STO \| \| STO \| \| STO \| \| ILE \| \| ILE \| \| ILE \| \| ILE \| \| ILE \| \| ILE \| \| ILE \| \| ILE \| \| ILE \| \| ILE \| \| ILE \| \| ILE \| \| ILE \| \| ILE \| \| ILE \| \| ILE \| \| ILE \| \| ILE \| \| ILE \| \| ILE \| | \| -0,82 \| \| --- \| \| 5,00 \| \| -1,87 \| \| 0,08 \| \| -0,38 \| \| -1,36 \| \| 1,21 \| \| -3,03 \| \| -1,87 \| \| -0,47 \| \| -2,40 \| \| -1,36 \| \| -1,24 \| \| 1,48 \| \| -0,46 \| \| -1,68 \| \| -0,59 \| \| 0,01 \| \| 0,07 \| \| 1,36 \| \| -1,37 \| \| -1,30 \| \| 0,01 \| \| 1,92 \| \| 2,33 \| \| 0,11 \| \| -1,30 \| \| 1,25 \| \| -0,98 \| \| -0,55 \| \| -2,05 \| \| -1,55 \| | \| 2,43 \| \| --- \| \| -0,47 \| \| 0,06 \| \| 3,54 \| \| 0,79 \| \| 0,87 \| \| 1,09 \| \| 0,69 \| \| 0,06 \| \| 1,65 \| \| -0,65 \| \| 0,87 \| \| -0,39 \| \| -6,78 \| \| -6,33 \| \| -6,70 \| \| -5,65 \| \| 0,71 \| \| -6,49 \| \| -6,58 \| \| -6,90 \| \| -6,39 \| \| 0,03 \| \| 0,02 \| \| -2,19 \| \| -6,06 \| \| -6,39 \| \| 0,03 \| \| -6,53 \| \| -5,87 \| \| -7,63 \| \| -6,09 \| | \| -0,19 \| \| --- \| \| 0,89 \| \| -0,79 \| \| 1,04 \| \| 1,86 \| \| 1,28 \| \| 0,28 \| \| 0,08 \| \| -0,79 \| \| -0,27 \| \| 0,08 \| \| 1,28 \| \| -0,63 \| \| -2,27 \| \| -1,85 \| \| -2,04 \| \| -1,58 \| \| 0,91 \| \| -1,46 \| \| -2,00 \| \| -2,95 \| \| -1,92 \| \| 0,91 \| \| -1,01 \| \| 0,94 \| \| -2,32 \| \| -1,92 \| \| 0,43 \| \| -2,75 \| \| -1,40 \| \| -1,77 \| \| -7,10 \| | \| -0,39 \| \| --- \| \| 0,99 \| \| 1,27 \| \| 0,96 \| \| 0,22 \| \| 0,23 \| \| -0,12 \| \| -1,73 \| \| 1,27 \| \| -0,35 \| \| -0,83 \| \| 0,23 \| \| -0,95 \| \| -2,64 \| \| -2,89 \| \| -3,75 \| \| -2,44 \| \| 0,76 \| \| -3,18 \| \| -3,08 \| \| -3,83 \| \| -3,51 \| \| 0,76 \| \| -2,55 \| \| 1,00 \| \| -1,04 \| \| -3,51 \| \| 0,26 \| \| -2,01 \| \| -2,74 \| \| -2,41 \| \| -2,55 \| | \| -0,19 \| \| --- \| \| 1,73 \| \| 1,24 \| \| 1,04 \| \| 1,18 \| \| -0,20 \| \| 0,28 \| \| 0,91 \| \| 1,24 \| \| -0,27 \| \| 1,18 \| \| -0,20 \| \| -0,63 \| \| -2,78 \| \| -2,84 \| \| -2,56 \| \| -1,58 \| \| 0,91 \| \| -1,33 \| \| -1,60 \| \| -2,56 \| \| -1,73 \| \| 0,91 \| \| -0,51 \| \| 1,80 \| \| -0,65 \| \| -1,73 \| \| 0,43 \| \| -0,91 \| \| -0,54 \| \| -1,17 \| \| -2,55 \| | \| 0,04 \| \| --- \| \| 2,32 \| \| 0,62 \| \| -0,04 \| \| 3,19 \| \| 4,49 \| \| -0,30 \| \| 1,81 \| \| 0,62 \| \| 0,36 \| \| -0,75 \| \| 4,49 \| \| -0,58 \| \| -3,02 \| \| -6,35 \| \| -5,58 \| \| -5,54 \| \| 0,53 \| \| -1,11 \| \| -0,58 \| \| -1,26 \| \| -1,98 \| \| 0,53 \| \| 0,37 \| \| 2,32 \| \| -1,07 \| \| -1,98 \| \| 0,37 \| \| -3,12 \| \| -1,46 \| \| -1,91 \| \| -2,64 \| | \| 2,10 \| \| --- \| \| 0,54 \| \| 0,47 \| \| -1,61 \| \| 0,61 \| \| -0,01 \| \| 1,76 \| \| -2,26 \| \| 0,47 \| \| -2,19 \| \| 0,75 \| \| -0,01 \| \| -0,11 \| \| -4,87 \| \| -7,22 \| \| -7,57 \| \| -4,29 \| \| -0,24 \| \| -6,87 \| \| -4,93 \| \| -7,89 \| \| -7,38 \| \| -0,24 \| \| -4,54 \| \| 0,78 \| \| -3,59 \| \| -7,38 \| \| 0,43 \| \| -4,78 \| \| -6,61 \| \| -4,92 \| \| -3,68 \| | \| 0,14 \| \| --- \| \| 0,83 \| \| 0,44 \| \| 0,81 \| \| 0,07 \| \| 0,48 \| \| -0,45 \| \| -1,85 \| \| 0,47 \| \| -0,42 \| \| -2,61 \| \| 1,71 \| \| -0,66 \| \| -2,59 \| \| -2,88 \| \| -2,42 \| \| -1,66 \| \| 0,46 \| \| -1,62 \| \| -1,10 \| \| -2,94 \| \| -3,17 \| \| 0,46 \| \| -1,58 \| \| 1,18 \| \| -0,82 \| \| -3,17 \| \| 0,23 \| \| -1,67 \| \| -1,19 \| \| -1,25 \| \| -1,65 \| |

**Table S4. Log_2_ fold changes in virulence genes expression in the M-SHIME.** ILE: ileal effluents; ASC: Ascending colon.

| **Donor** | **Hours post-infection** | | **Gut Region** | ***eltB*** | ***estP*** | ***leoA*** | ***tolC*** | ***fimH*** | ***tia*** |
| --- | --- | --- | --- | --- | --- | --- | --- | --- | --- |
| \| 1 \| \| --- \| \| 2 \| \| 3 \| \| 4 \| \| 5 \| \| 6 \| \| 1 \| \| 2 \| \| 3 \| \| 4 \| \| 5 \| \| 6 \| \| 1 \| \| 2 \| \| 3 \| \| 4 \| \| 5 \| \| 6 \| \| 1 \| \| 2 \| \| 3 \| \| 4 \| \| 5 \| \| 6 \| \| 1 \| \| 2 \| \| 3 \| \| 4 \| \| 5 \| \| 6 \| \| 1 \| \| 2 \| \| 3 \| \| 4 \| \| 5 \| \| 6 \| | \| 1 \| \| --- \| \| 1 \| \| 1 \| \| 1 \| \| 1 \| \| 1 \| \| 3 \| \| 3 \| \| 3 \| \| 3 \| \| 3 \| \| 3 \| \| 27 \| \| 27 \| \| 27 \| \| 27 \| \| 27 \| \| 27 \| \| 5 \| \| 5 \| \| 5 \| \| 5 \| \| 5 \| \| 5 \| \| 20 \| \| 20 \| \| 20 \| \| 20 \| \| 20 \| \| 20 \| \| 29 \| \| 29 \| \| 29 \| \| 29 \| \| 29 \| \| 29 \| | \| ILE \| \| --- \| \| ILE \| \| ILE \| \| ILE \| \| ILE \| \| ILE \| \| ILE \| \| ILE \| \| ILE \| \| ILE \| \| ILE \| \| ILE \| \| ILE \| \| ILE \| \| ILE \| \| ILE \| \| ILE \| \| ILE \| \| ASC \| \| ASC \| \| ASC \| \| ASC \| \| ASC \| \| ASC \| \| ASC \| \| ASC \| \| ASC \| \| ASC \| \| ASC \| \| ASC \| \| ASC \| \| ASC \| \| ASC \| \| ASC \| \| ASC \| \| ASC \| | | \| -0.43 \| \| --- \| \| -0.74 \| \| 1.17 \| \| -0.82 \| \| 0.82 \| \| -0.92 \| \| -2.91 \| \| -2.25 \| \| 3.94 \| \| -3.23 \| \| -5.45 \| \| -5.58 \| \| -9.99 \| \| -12.25 \| \| -10.44 \| \| -7.86 \| \| -11.70 \| \| -8.29 \| \| -2.76 \| \| 2.77 \| \| 2.22 \| \| 2.49 \| \| 6.88 \| \| 3.36 \| \| -4.28 \| \| -5.44 \| \| -5.36 \| \| -2.56 \| \| -0.46 \| \| -5.30 \| \| -4.11 \| \| -6.92 \| \| -13.29 \| \| -16.61 \| \| -19.95 \| \| -19.93 \| | \| 0.81 \| \| --- \| \| -1.02 \| \| 0.21 \| \| -0.26 \| \| 0.26 \| \| -1.70 \| \| 2.30 \| \| 2.68 \| \| 1.67 \| \| 0.83 \| \| -1.08 \| \| -1.84 \| \| -13.29 \| \| -17.61 \| \| -11.48 \| \| -13.28 \| \| -8.43 \| \| -10.57 \| \| 3.72 \| \| 3.58 \| \| 2.24 \| \| -13.27 \| \| 0.56 \| \| 0.91 \| \| 4.00 \| \| 2.71 \| \| 2.26 \| \| -13.29 \| \| -13.21 \| \| -13.25 \| \| -13.19 \| \| 0.82 \| \| -12.27 \| \| -13.29 \| \| -12.47 \| \| -12.68 \| | \| 3.00 \| \| --- \| \| -1.83 \| \| -1.17 \| \| -0.86 \| \| 0.86 \| \| 0.91 \| \| 0.81 \| \| 2.19 \| \| 1.51 \| \| -2.31 \| \| -3.10 \| \| -2.83 \| \| -9.72 \| \| -13.29 \| \| -13.81 \| \| -11.29 \| \| -11.58 \| \| -13.22 \| \| -2.98 \| \| -13.29 \| \| -5.95 \| \| -1.34 \| \| -3.99 \| \| -3.22 \| \| 1.43 \| \| 0.55 \| \| -13.29 \| \| -2.32 \| \| -13.29 \| \| -4.91 \| \| -13.29 \| \| -1.49 \| \| -16.34 \| \| -14.49 \| \| -19.29 \| \| -11.29 \| | \| 1.47 \| \| --- \| \| -1.85 \| \| -0.13 \| \| -0.68 \| \| 0.68 \| \| 0.46 \| \| -1.24 \| \| -1.25 \| \| 0.31 \| \| 0.46 \| \| 0.97 \| \| 0.65 \| \| 0.95 \| \| 0.09 \| \| 0.35 \| \| -0.45 \| \| -2.07 \| \| -11.29 \| \| 0.18 \| \| 1.79 \| \| -0.24 \| \| 1.78 \| \| 0.37 \| \| 1.13 \| \| 0.19 \| \| 0.01 \| \| 0.23 \| \| 1.06 \| \| -0.02 \| \| -0.39 \| \| 1.06 \| \| 0.38 \| \| -13.33 \| \| -13.24 \| \| -13.78 \| \| -13.29 \| | \| -1.62 \| \| --- \| \| 0.73 \| \| 0.89 \| \| -0.29 \| \| 0.29 \| \| 0.31 \| \| 0.19 \| \| 0.36 \| \| -2.54 \| \| -3.20 \| \| -1.83 \| \| -2.06 \| \| -3.01 \| \| -5.67 \| \| -4.53 \| \| -2.72 \| \| -2.55 \| \| -14.00 \| \| -0.02 \| \| 0.25 \| \| -2.17 \| \| 1.37 \| \| -4.27 \| \| -3.09 \| \| -1.16 \| \| -0.35 \| \| -0.85 \| \| -1.84 \| \| -2.56 \| \| -3.92 \| \| -2.15 \| \| -0.75 \| \| -11.46 \| \| -13.29 \| \| -13.67 \| \| -13.80 \| | \| -0.53 \| \| --- \| \| -0.64 \| \| 1.17 \| \| -13.29 \| \| -13.38 \| \| -0.74 \| \| -2.61 \| \| -2.50 \| \| 0.14 \| \| -13.33 \| \| -13.09 \| \| -13.29 \| \| -13.12 \| \| -12.29 \| \| -5.73 \| \| -11.48 \| \| -12.77 \| \| -13.29 \| \| -13.38 \| \| -13.02 \| \| -8.08 \| \| -11.28 \| \| -11.29 \| \| -13.44 \| \| -13.29 \| \| -13.29 \| \| -12.24 \| \| -9.72 \| \| -13.80 \| \| -13.01 \| \| -12.44 \| \| -11.96 \| \| -13.33 \| \| -11.28 \| \| -13.29 \| \| -13.66 \| |

**Table S5. Parameters of the TIM-1 and M-SHIME systems.** A) When simulating digestive conditions of a healthy adult after intake of a glass of water in the TIM-1. T_1/2_ represents the half-time of gastric and ileal deliveries. B) When simulating a cycle of digestive and fermentative conditions of a healthy adult in the M-SHIME. The cycle of entering and emptying secretions is repeated 3 times per day in order to physiologically reproduce meals intake.

|  | TIM-1 | | | | M-SHIME | | | |
| --- | --- | --- | --- | --- | --- | --- | --- | --- |
| Parameters of *in vitro* digestion of a glass of water | Gastric compartment | Duodenal compartment | Jejunal compartment | Ileal  Compartment | Gastric compartment | Duodenal-jejunal compartment | Ileal compartment | Ascending colon  compartment |
| pH | from 6 (T0) to 1.5 (90 to 300 min) | maintained at 6.4 | maintained at 6.9 | maintained at 7.2 | 2 | 5 | 6 | maintained at  6.2-6.4 |
| Volume (mL) | 200  (initial) | 30 | 115 | 115 | 140 | 200 | 200 | 500 to 700 |
| Secretions | (**i**) 130 U min^-1^ of pepsin  (**ii**) 5 U min^-1^ of lipase  (**iii**) HCl 0.3 M | (**i**) 20 mg min^-1^ of bile salts 27.9 mM (first 30 min of digestion) then 10 mg min^-1^ of bile salts 9.3 mM  (**ii**) 20 mg min^-1^ of pancreatic juice 4 USP  (**iii**) Trypsin 2 mg min^-1^  (**iv**) NaHCO_3_ 0.5 M if necessary | (**i**) NaHCO_3_ 0.5 M if necessary | (**i**) NaHCO_3_ 0.5 M if necessary | (**i**) 14 ml min^-1^ of nutritional medium  (**ii**) HCl 0.5 M | (**i**) 12 ml min^-1^ of pancreatic juice and bile salts 0.01 mM  (**ii**) NaHCO_3_  0.5 M | (**i**) NaOH 0.5 M if necessary | (**i**) NaOH 0.5 M if necessary |
| Half-emptying time (min) / Residence time  (h) | T_1/2_ = 15 min | - | - | T_1/2_ = 150 min | 1 h | 2h30 | 3 h | 20 h |
| Chyme mixing | water pressure | water pressure | water pressure | water pressure | magnetic stirrer | magnetic stirrer | magnetic stirrer | magnetic stirrer |
| Absorption | - | - | yes | yes | - | - | - | - |
| [Total microbes] | sterile | sterile | sterile | sterile | sterile | sterile | 9-10 log_10_ | 11-12 log_10_ |
| Mucosal phase | - | - | - | - | - | - | yes | yes |
| Oxygen level (%) | 20 | 20 | 20 | 20 | < 3 | < 3 | < 3 | < 3 |
| Temperature (°C) | 37 | 37 | 37 | 37 | 37 | 37 | 37 | 37 |

**Table S6. General characteristic of the fecal donors involved in the M-SHIME experiments.**

|  | **Donor 1** | **Donor 2** | **Donor 3** | **Donor 4** | **Donor 5** | **Donor 6** |
| --- | --- | --- | --- | --- | --- | --- |
| **Gender** | female | female | male | male | female | Male |
| **Age** | 27 | 28 | 32 | 36 | 25 | 29 |
| **Nationality** | French | Turkish | Belgian | African | Belgian | Belgian |

**Table S7. Static *in vitro* gastro-jejunal digestion procedure.** Prior to introduction of the pathogen in the SHIME ileum, ETEC was pre-digested in a static batch incubation (Erlenmeyer), to reproduce the physicochemical parameters of a gastro-jejunal digestion found in the TIM-1 system. Secretions were manually added and pH was manually controlled during the 180 min digestion.

| Parameters of static *in vitro* digestion | Gastric compartment | Duodenum-Jejunum |
| --- | --- | --- |
| pH | from 6 (T0) to 2.1 | maintained at 6.8 |
| Volume (mL) | 50 | 90 |
| Secretions | (**i**) 5.36 mg pepsin (727 U mg^-1^)  (**ii**) 4.28 mg lipase (32 U mg^-1^)  (**iii**) HCl 0.3 M  (**iv**) NaHCO_3_ 0.5 M if necessary | (**i**) 0.9 g bile salts (27.9 mM in solution)  (**ii**) 1.8 g of pancreatin 4 USP  (**iii**) Trypsin 2 mg mL^-1^  (**iv**) NaHCO_3_ 0.5 M if necessary |
| Time period in batch (min) | 30 | 150 |
| Chyme mixing | magnetic stirrer | magnetic stirrer |
| [Total microbes] | sterile | sterile |
| Oxygen level (%) | 20 | 20 |
| Temperature (°C) | 37  (incubator) | 37  (incubator) |

**Table S8. ETEC primers used in the study.**

| **Gene** | **Target** | **Primer sequence 5’-3’** | **Amplicon length (pb)** | **SI References** |
| --- | --- | --- | --- | --- |
| **Total Flora** | | | | |
| *16S* | 16S | F- ACTCCTACGGGAGGCAGCAG  R- ATTACCGCGGCTGCTGG | 198 | [54] |
| **Genes to monitor ETEC by qPCR** | | | | |
| *gspD* | Type II secretion system for LT delivery | F- CGGATACCAACGGCGATCT  R- CCGCTAAAGCCGGAAAGAA | 55 | [50] |
| *16S*  *rRNA* | 16S *Enterobacteriaceae* | F- CATGCCGCGTGTATGAAGAA  R- CGGGTAACGTCAATGAGCAAA | 96 | [51] |
| **Virulence genes for RT-qPCR** | | | | |
| *eltB* | LT toxin | F- GGCAGGCAAAAGAGAAATGG  R- TCCTTCATCCTTTCAATGGCT | 117 | [53] |
| *estP* | ST toxin | F- TCTTTCCCCTCTTTTAGTCAG  R- ACAGGCAGGATTACAACAAAG | 165 | [55] |
| *leoA* | Labile enterotoxin output | F- AAACGGTGCATATCCTCGTC  R- AAATGCTGCCACCGAAATAC | 168 | This study |
| *tolC* | TolC outer membrane protein | F- AAGCCGAAAAACGCAACCT  R- CAGAGTCGGTAAGTGACCATC | 101 | [56] |
| *cfa/Ib* | CFA/Ib Adhesin | F- TCAGTGTGTCATGGGGAGG  R- CAGTTTTAGGTGCAGCGCTA | 138 | This study |
| *tia* | Tia Adhesin | F- ACAGGCTTTTATGTGACCGGTAA  R- GACGGAAGCGCTGGTCAGT | 67 | [59] |
| *fimH* | Minor component of Type I pili | F- GTGCCAATTCCTCTTACCGTT  R- TGGAATAATCGTACCGTTGCG | 164 | [57] |
| *rpoS* | Sigma 38 protein | F- GCGCGGTAGAGAAGTTTGAC  R- GGCTTATCCAGTTGCTCTGC | 229 | [58] |
| **Reference genes for RT-qPCR** | | | | |
| *arcA* | Aerobic respiration control | F- GTTCTTACCCGGCAGATTGA  R- CAGACCCCGCACATTCTTAT | 180 | This study |
| *gapA* | *E. coli* GAPDH | F- CGTTGAAGTGAAAGACGGTCATC  R- CAACACCAACTTCGTCCCATTT | 101 | [59] |

CFA: Colonization factor antigen, F: Forward, GAPDH: Glyceraldehyde 3-phosphate dehydrogenase, LT: Heat-labile enterotoxin, R: Reverse, ST: Heat-stable enterotoxin.

**Table S9. RDP Seqmatch and NCBI BLAST results for the most abundant species and/or species of interest in the M-SHIME.** The similarity score (Sab), as calculated by RDP, and the NCBI BLAST output for the best hit and next best hit(s) is shown. The NCBI maximal score (not shown) equalled the total score for all displayed entries. Indicated in bold, the OTU numbers for which they have been replaced by the species names for the microbial analysis. NA= Not Available.

|  |  | **RDP** | **NCBI BLAST** | | | |
| --- | --- | --- | --- | --- | --- | --- |
| **OTU** | **Species** | **Sab** | **Total score** | **Query coverage (%)** | **E-value** | **Identity (%)** |
| **1** | *Escherichia/Shigella fergusonii*  *Escherichia/Shigella flexneri*  *Shigella sonei*  *Escherichia coli*  *Escherichia vulneris* | 1.000  1.000  1.000  1.000  1.000 | 787  787  787  787  787 | 100  100  100  100  100 | 0.0  0.0  0.0  0.0  0.0 | 100  100  100  100  100 |
| **2** | *Anaerovibrio lipolyticus*  *Selenomonas bovis* | 0.771  0.752 | 638  614 | 99  100 | 0.0  1e-175 | 94  93 |
| **3** | ***Klebsiella pneumoniae***  *Klebsiella quasipneumoniae*  *Klebsiella variicola*  *Serratia liquefaciens* | **0.969**  0.969  0.964  NA | **782**  776  NA  776 | **100**  100  NA  100 | **0.0**  0.0  NA  0.0 | **99**  99  NA  99 |
| **4** | *Leclercia adecarboxylata*  *Enterobacter cloacae*  *Enterobacter ludwigii*  *Pantoea agglomerans*  *Enterobacter kobei*  *Salmonella enterica* | 1.000  0.983  1.000  NA  0.983  0.983 | 787  787  787  787  784  782 | 100  100  100  100  100  100 | 0.0  0.0  0.0  0.0  0.0  0.0 | 100  100  100  99  99  99 |
| **5** | ***Mitsuokella multacida***  *Mitsuokella jalaludinii*  *Selenomonas bovis* | **0.983**  0.954  0.814 | **784**  754  630 | **100**  100  99 | **0.0**  0.0  1e-180 | **99**  99  93 |
| **6** | ***Faecalibacterium prausnitzii***  *Gemmiger formicilis* | **0.982**  0.716 | **736**  569 | **100**  100 | **0.0**  7e-159 | **99**  92 |
| **7** | *Succinivibrio dextrinosolvens*  *Anaerobiospirillum succiniciproducens* | 0.919  0.646 | 688  540 | 100  100 | 0.0  2e-153 | 98  91 |
| **8** | ***Clostridium bolteae***  *Clostridium asparagiforme* | **1.000**  0.923 | **741**  986 | **100**  100 | **0.0**  0.0 | **100**  98 |
| **9** | ***Veillonella dispar***  *Veillonella tobetsuensis*  *Veillonella parvula* | **0.968**  0.949  0.929 | **776**  760  754 | **100**  100  100 | **0.0**  0.0  0.0 | **99**  99  99 |
| **10** | ***Bacteroides fragilis***  *Bacteroides ovatus* | **1.000**  NA | **778**  649 | **100**  100 | **0.0**  0.0 | **100**  95 |
| **11** | *Mitsuokella jalaludinii*  *Mitsuokella multacida* | 0.983  0.937 | 782  750 | 100  100 | 0.0  0.0 | 99  98 |
| **12** | ***Bacteroides dorei***  *Bacteroides vulgatus* | **1.000**  0.951 | **778**  756 | **100**  100 | **0.0**  0.0 | **100**  99 |
| **13** | ***Pseudomonas aeruginosa***  *Pseudomonas guezennei* | **1.000**  NA | **787**  776 | **100**  100 | **0.0**  0.0 | **100**  99 |
| **14** | ***Faecalibacterium prausnitzii***  *Gemmiger formicilis* | **0.918**  0.735 | **713**  592 | **100**  100 | **0.0**  7e-169 | **99**  93 |
| **15** | ***Bacteroides thetaiotaomicron***  *Bacteroides faecichinchillae*  *Bacteroides faecis* | **1.000**  0.947  0.935 | **778**  756  739 | **100**  100  100 | **0.0**  0.0  0.0 | **100**  99  98 |
| **16** | ***Bacteroides uniformis***  *Bacteroides rodentium* | **1.000**  0.906 | **778**  717 | **100**  100 | **0.0**  0.0 | **100**  97 |
| **17** | *Acidaminococcus fermentans*  *Acidaminococcus intestini* | 0.925  NA | 737  671 | 100  100 | 0.0  0.0 | 98  95 |
| **18** | ***Clostridium butyricum***  *Clostridium saccharobutylicum* | **1.000**  0.941 | **741**  719 | **100**  100 | **0.0**  0.0 | **100**  99 |
| **19** | *Citrobacter freundii*  *Citrobacter brakii* | 0.990  0.971 | 782  771 | 100  100 | 0.0  0.0 | 99  99 |
| **20** | ***Acidaminococcus intestine***  *Acidaminococcus fermentans* | **1.000**  0.885 | **787**  704 | **100**  100 | **0.0**  0.0 | **100**  96 |
| **21** | ***Gemmiger formicilis***  *Subdoligranulum variabile* | **1.000**  0.959 | **741**  719 | **100**  100 | **0.0**  0.0 | **100**  99 |
| **22** | *Bacteroides timonensis*  *Bacteroides cellulosilyticus*  *Bacteroides intestinalis* | NA  0.934  0.888 | 761  750  739 | 100  100  100 | 0.0  0.0  0.0 | 99  99  98 |
| **23** | ***Eubacterium rectale***  *Roseburia faecis*  *Roseburia intestinalis* | **0.949**  0.941  0.892 | **741**  713  939 | **100**  100  100 | **0.0**  0.0  0.0 | **100**  99  98 |
| **24** | ***Parabacteroides distasonis***  *Parabacteroides gordonii*  *Parabacteroides faecis* | **0.971**  0.672  0.613 | **778**  756  601 | **100**  100  100 | **0.0**   \| 2e-174 \| \| --- \| \| 1e-171 \| | **100**  93  92 |
| **25** | *Bacteroides ovatus*  *Bacteroides xylanisolvens* | 0.978  NA | 773  730 | 100  100 | 0.0  0.0 | 99  98 |
| **26** | ***Phascolarctobacterium faecium***  *Phascolarctobacterium succinatutens* | **1.000**  0.734 | **787**  632 | **100**  100 | **0.0**  0.0 | **100**  93 |
| **27** | ***Clostridium perfringens***  *Eubacterium tarantellae* | **1.000**  NA | **741**  691 | **100**  100 | **0.0**  0.0 | **100**  98 |
| **28** | *Mitsuokella jalaludinii*  *Mitsuokella multacida*  *Selenomonas bovis* | 0.935  0.886  NA | 704  673  675 | 100  100  100 | 0.0  0.0  0.0 | 96  95  95 |
| **29** | *Bilophila wadsworthia*  *Desulfovibrio simplex* | 0.973  NA | NA  604 | NA  100 | NA  9e-173 | NA  92 |
| **30** | ***Blautia faecis***  *Blautia glucerasea* | **1.000**  0.926 | **741**  726 | **100**  100 | **0.0**  0.0 | **100**  99 |
| **35** | ***Bifidobacterium longum*** | **0.985** | **750** | **100** | **0.0** | **100** |
|  | *Bifidobacterium breve* | 0.918 | NA | NA | NA | NA |
|  |  |  |  |  |  |  |
| **40** | *Prevotella copri*  *Prevotella oulorum* | 0.851  0.751 | 717  640 | 100  100 | 0.0  0.0 | 97  94 |
| **53** | ***Marivita hallyeonensis***  *Marivita roseacus* | **0.964**  0.940 | **730**  719 | **100**  100 | **0.0**  0.0 | **99**  99 |
| **55** | *Enterococcus hirae*  *Enterococcus villorum*  *Enterococcus ratti*  *Enterococcus durans* | 1.000  1.000  1.000  1.000 | 787  787  787  787 | 100  100  100  100 | 0.0  0.0  0.0  0.0 | 100  100  100  100 |
| **90** | ***Clostridium scindens***  *Dorea longicatena* | **1.000**  NA | **741**  669 | **100**  100 | **0.0**  0.0 | **100**  97 |
| **115** | ***Klebsiella variicola***  *Klebsiella pneumonia* | **0.983**  0.959 | **782**  776 | **100**  100 | **0.0**  0.0 | **99**  99 |
| **120** | *Oscillibacter valericigenes*  *Oscillibacter ruminantium* | 0.778  0.745 | 617  606 | 100  100 | 1e-176  2e-173 | 94  94 |
| **124** | ***Blautia luti***  *Blautia stercoris* | **0.987**  0.926 | **736**  691 | **100**  100 | **0.0**  0.0 | **99**  98 |
| **151** | ***Bifidobacterium angulatum***  *Bifidobacterium merycicum* | **1.000**  0.974 | **754**  737 | **100**  99 | **0.0**  0.0 | **100**  99 |
| **156** | *Barnesiella viscericola*  *Barnesiella intestinihominis* | 0.571  0.623 | -  - | -  - | -  - | -  - |
| **175** | *Blautia coccoides*  *Muricome intestine*  *Clostridium oroticum* | 0.867  0.824  0.810 | 680  675  669 | 100  100  100 | 0.0  0.0  0.0 | 97  97  97 |
| **201** | *Mycobacterium moriokaense*  *Mycobacterium grossiae*  *Mycobacterium aquaticum* | 0.915  NA  NA | 701  701  701 | 100  100  100 | 0.0  0.0  0.0 | 98  98  98 |
| **250** | *Schlegelella thermodepolymerans*  *Zhizhongheella caldifontis* | 0.763  0.741 | -  - | -  - | -  - | -  - |
| **275** | ***Bacillus xiaoxiensis*** | **1.000** | **766** | **100** | **0.0** | **100** |
| **314** | ***Barnesiella intestinihominis***  *Barnesiella viscericola* | **0.971**  0.791 | **767**  684 | **100**  100 | **0.0**  0.0 | **99.5**  95.9 |
| **429** | *Sutterella stercoricanis* | 0.928 | 749 | 100 | 0.0 | 98 |
